# Adverse drug reactions triggered by the common HLA-B*57:01 variant: virtual screening of DrugBank using 3D molecular docking

**DOI:** 10.1186/s13321-018-0257-z

**Published:** 2018-01-30

**Authors:** George Van Den Driessche, Denis Fourches

**Affiliations:** Department of Chemistry, Bioinformatics Research Center, North Carolina State University, Raleigh, NC USA

**Keywords:** Molecular docking, Virtual screening, HLA, ADR, HLA-B*57:01, Abacavir, DrugBank

## Abstract

**Background:**

Idiosyncratic adverse drug reactions have been linked to a drug’s ability to bind with a human leukocyte antigen (HLA) protein. However, due to the thousands of HLA variants and limited structural data for drug-HLA complexes, predicting a specific drug-HLA combination represents a significant challenge. Recently, we investigated the binding mode of abacavir with the HLA-B*57:01 variant using molecular docking. Herein, we developed a new ensemble screening workflow involving three X-ray crystal derived docking procedures to screen the DrugBank database and identify potentially HLA-B*57:01 liable drugs. Then, we compared our workflow’s performance with another model recently developed by Metushi et al., which proposed seven in silico HLA-B*57:01 actives, but were later found to be experimentally inactive.

**Methods:**

After curation, there were over 6000 approved and experimental drugs remaining in DrugBank for docking using Schrodinger’s GLIDE SP and XP scoring functions. Docking was performed with our new consensus-like ensemble workflow, relying on three different X-ray crystals (3VRI, 3VRJ, and 3UPR) in presence and absence of co-binding peptides. The binding modes of HLA-B*57:01 hit compounds for all three peptides were further explored using 3D interaction fingerprints and hierarchical clustering.

**Results:**

The screening resulted in 22 hit compounds forecasted to bind HLA-B*57:01 in all docking conditions (SP and XP with and without peptides P1, P2, and P3). These 22 compounds afforded 2D-Tanimoto similarities being less than 0.6 when compared to the structure of native abacavir, whereas their 3D binding mode similarities varied in a broader range (0.2–0.8). Hierarchical clustering using a Ward Linkage revealed different clustering patterns for each co-binding peptide. When we docked Metushi et al.’s seven proposed hits using our workflow, our screening platform identified six out of seven as being inactive. Molecular dynamic simulations were used to explore the stability of abacavir and acyclovir in complex with peptide P3.

**Conclusions:**

This study reports on the extensive docking of the DrugBank database and the 22 HLA-B*57:01 liable candidates we identified. Importantly, comparisons between this study and the one by Metushi et al. highlighted new critical and complementary knowledge for the development of future HLA-specific in silico models.

**Electronic supplementary material:**

The online version of this article (10.1186/s13321-018-0257-z) contains supplementary material, which is available to authorized users.

## Background

Adverse drug reaction (ADR) was defined by the World Health Organization (WHO) in 1970 as “a response to a drug that is noxious and unintended and occurs at doses normally used in man for the prophylaxis, diagnosis or therapy of disease, or for modification of physiological function” [1]. ADRs are now classified into two basic categories: Type A (*predictable*) and Type B (*idiosyncratic*) [2–5]. Predictable ADR events are directly caused by drugs’ polypharmacology and typically show a dose-dependent relationship; however, idiosyncratic ADRs are not dependent upon drug pharmacology and/or dose [2–5]. Idiosyncratic ADRs can be further divided into immune-mediated or non-immune mediated (metabolic idiosyncrasy) [4].

Immune-mediated ADRs are rarely observed during clinical trials and are extremely challenging to forecast. Little is known about the exact mechanisms of actions initiating and propagating such type of ADRs. Importantly, advances in the field of pharmacogenomics have greatly increased our ability to prevent ADR events by determining patients’ genetic markers [6, 7]. There are indeed several genetic markers associated with a drug’s ability to cause ADR events: for instance, drug metabolizing proteins (cytochrome P450, CYP, glucose-6-phosphate dehydrogenase, G6PD, nucleoside diphosphate linked moiety X-type motif 15, NUDT15), drug transporter proteins (ATP-binding cassette, ABC, solute carrier organic anion transporter family, SLCO1B1) or antigen-presenting cells, APC (human leukocyte antigen, HLA) [7]. Recently, significant associations between human leukocyte antigen (HLA) proteins and idiosyncratic ADRs have been identified [4–10].

Interestingly, HLA-mediated ADR events are less understood due to a variety of reasons. First, according to the IMGT/HLA database (http://www.ebi.ac.uk/ipd/imgt/hla/), there are over 16,000 variants of HLA that have been reported so far [11]. Second, those variants occur at different frequencies in the general population. For example, a study conducted by Cao et al. [12] determined that the HLA-B*15:02 variant was only found in 0.2% of African-Americans and 4.9% of Asian patients, whereas it was unobserved in Caucasians, Hispanics, and North American Natives. Meanwhile, when Cao et al. studied the HLA-A*31:01 variant, they found that this variant occurred in 0.8% of African-Americans, 3.1% of Asians, 3.2% of Caucasians, 4.9% of Hispanics, and up to 7.8% of North American Natives who participated in the study [12]. The changing frequency of HLA alleles in the general population results in a third challenging observation: binding promiscuity between HLA variants and drugs. For example, Chung et al. identified a correlation between carbamazepine and the HLA-B*15:02 variant in a population of Han-Chinese patients who were suffering from Steven–Johnson Syndrome (SJS), an ADR event causing serious skin rashes [13]. Recently, Genin et al. identified another correlation between carbamazepine and the HLA-A*31:01 variant in Norther Europeans who were suffering from SJS as well [14]. Binding promiscuity is not only observed in drugs binding multiple HLA-variants, but also in HLA-variants binding multiple drugs. Perhaps the most well-known example to date is HLA-B*57:01, which has been identified to bind with three drugs: abacavir which can cause the abacavir hypersensitivity syndrome (AHS), and flucloxacillin and pazopanib, which both cause drug induced liver injury (DILI) [8, 9, 15–18]. There may also be a third type of binding promiscuity in HLA-complexes: peptide binding promiscuity. To date, there are 17 crystal structure depositories of the HLA-B*57:01 variant in the PDB with four crystal structures containing abacavir and a unique co-binding peptide (PDB: 3VRI, 3VRJ, 3UPR, and 5U98), there are seven crystal structures of HLA-B*57:01 with a co-binding peptide (PDB: 2RFX, 3X11, 3X12, 5T5M, 5T6W, 5T6X, and 5T6Y), and six crystal structures of HLA-B*57:01 with co-binding peptide complexed to a T cell (PDB: 3WUW, 3VH8, 5B38, 5B39, 5T70, and 5T6Z [15, 16, 19–26]. Notably, of these 17 crystal structures there are nine unique co-binding peptides indicating that when studying HLA-complexes one needs to consider HLA-, drug-, and peptide-binding promiscuity. Other examples of HLA-drug associations include the drug allopurinol which has been reported to cause SJS in patients with the HLA-B*58:01 variant [27, 28]. Furthermore, HLA-bound drugs are believed to occur through three different mechanisms via an altered repertoire complex, a pharmacological interaction (p.i.) complex, or a hapten complex [5, 29, 30]. Clearly, due to the high number of HLA variants, their population-specific frequency, drug promiscuity towards HLA binding (or vice versa), and numerous binding mechanisms the prediction of HLA-induced ADR events represents a serious challenge.

In such context, the use of in silico modeling and screening techniques can provide great insight and guidance, especially when it comes to (1) identifying potential HLA binders among very large libraries of chemicals, (2) prioritizing those predicted top binders for experimental confirmation, and (3) understanding the molecular interactions those chemicals can form once docked in the HLA antigen-presenting pocket. Two research groups recently utilized such in silico techniques by employing 3D molecular docking to study the binding mode abacavir with HLA-B*57:01 and carbamazepine with HLA-B*15:02. Notably, Ostrov et al. [16] confirmed abacavir’s binding mode with HLA-B*57:01 via X-ray crystallization (PDB: 3UPR); contrarily, Illing et al. [15] used two X-ray crystals of abacavir and HLA-B*57:01 (PDB: 3VRI, 3VRJ) to test a postermolecular docking’s reliability prior to docking the interaction between carbamazepine and HLA-B*15:02. However, in the absence of extensive experimental structural data, computational tools can still provide great insights. For example, the binding interactions of HLA-B*58:01 with allopurinol, HLA-A*31:01 and HLA-B*15:02 with carbamazepine, HLA-B*14:02 with nevirapine, HLA-DRB1*07:01 with ximelagatran, and HLA-B*53:01 with raltegravir have all been studied through a combination of homology modeling and 3D molecular docking [31–38].

Specifically, the study by Wei et al. [33] resulted in the development of a homology model of the HLA-B*15:02 variant that was later used by Zhou et al. [34] to elucidate a possible pharmacological interaction (p. i.) complex binding mode of carbamazepine with the HLA-B*15:02 variant. Additionally, Zhou et al. were able to conduct molecular dynamic simulations (MDS) of the HLA-B*15:02-carbamazepine-T-cell signaling pathway [34]. A p. i. complex HLA signaling pathway occurs when a drug, or antigen, binds to the solvent exposed surface of the co-binding peptide [5, 30]; naturally, these types of interactions are relatively weak explaining why obtaining the crystal structure of such systems is extremely difficult.

The X-ray structures solved by Ostrov et al. [16] (PDB: 3UPR) and Illing et al. [15] (PDB: 3VRI and 3VRJ) provided the research community with a fully solved binding mode for abacavir in complex with HLA-B*57:01. These crystals also revealed that abacavir binds in an altered repertoire mechanism. Such altered repertoire binding mechanisms occur when the drug slightly displaces the co-binding peptide by binding with the HLA peptide binding pocket [5, 30]. Using these three crystals, modelers started developing computational models regarding such an altered repertoire binding mode. In 2015, Ho et al. [39] conducted a molecular docking study where they identified that flucloxacillin metabolites may also bind via an altered repertoire mechanism using the 3UPR X-ray crystal structure. Interestingly, flucloxacillin has also been proposed to bind via a hapten complex, through the formation of a covalent bond with either the co-binding peptide or HLA-B*57:01, as the presence of lysine residues have been shown to form covalent bonds with β-lactam chemical structures [40]. Another study by Yang et al. [41] cross-docked abacavir with HLA-B*57:01 and several other HLA-variants to determine abacavir’s ability to bind multiple variants. Unfortunately, these docking studies were performed without a co-binding peptide, even though the peptides have an impact on the binding conformation of abacavir. Recently, Metushi et al. [42] conducted a full in silico to in vitro screening of the ZINC database searching for activity at the HLA-B*57:01 variant. The authors used a combination of ligand-based screening and structure-based molecular docking to identify several compounds, with acyclovir predicted as most active, for experimental assays [42]. However, using a T-cell response based assay [43], it was determined that their predicted molecules were inactive towards HLA-B*57:01.

In the absence of extensive HLA-related chemogenomics data in the public domain, the development of virtual screening models that can accurately forecast drug-HLA interactions is extremely difficult. As we noted in our *proof*-*of*-*concept* study [44], there appears to be an inconsistent application of the molecular docking methodology when studying HLA systems. Indeed, the literature tends to be rather scarce regarding the pre-processing of HLA protein variants prior to modeling as well as which considerations (if any) were made with regards to the co-binding peptide and its ability to stabilize the bound drug. Even a recent study by Urban et al. [45] underlined the importance of taking into consideration the co-binding peptide in their analysis of HLA-B*35:02 and minocycline. Undoubtedly, the modeling of HLA-drug interactions is still in its infancy and the development of more insightful and predictive models is needed [46].

In our recent study [44], we explored the complex intermolecular interactions between the binding pocket of HLA-B*57:01, the co-binding drug abacavir, and three co-binding peptides from the three available X-ray crystals (PDB: 3VRI, 3VRJ, 3UPR) by Illing et al. [15] and Ostrov et al. [16]. After conducting structural alignments of the individual components of our system (HLA-B*57:01 peptide binding pocket, bound abacavir, and co-binding peptide), we concluded that the most significant differences between binding pocket, abacavir, and peptide occurred from the peptide amino acid sequence [44]. Performing a peptide backbone alignment revealed that the 3D-structure of the peptide backbone was highly conserved [44]. We also conducted molecular docking using Glide from the Schrodinger Suite to self-dock abacavir with and without the three co-binding peptides P1 (PDB: 3VRI), P2 (PDB: 3VRJ), and P3 (PDB: 3UPR). Interestingly, we found that the co-binding peptide provided ~ 2 kcal/mol of stabilization as shown by their respective Docking Score (DS) and also proceeded to conserve the binding mode orientation of abacavir [44]. When docking was performed without co-binding peptide, abacavir was observed in two stable binding modes, but when peptide was included in the docking procedure, there was only one stable binding mode remaining [44]. Next, we docked a small test set of predicted HLA-liable drugs including two HLA-B*57:01 actives: flucloxacillin and pazopanib [17, 18]. Interestingly, our model was unable to identify either drug as active [44]. This result was believed to occur from three possible reasons: (1) our model was built using X-ray crystals of abacavir in an altered repertoire binding mode causing our models to be biased towards drugs that have a highly similar binding orientation as abacavir (i.e., abacavir-specific), (2) our test set of compounds did not contain the HLA-liable metabolites of flucloxacillin or pazopanib, and (3) the binding affinity of these compounds could be peptide-specific [44].

Herein, using all these recent insights into modeling drug-HLA interactions, this new study aims at developing and testing an ensemble docking platform [44] to screen the entire DrugBank database for potentially HLA-B*57:01 liable compounds that are currently unknown and/or untested. At the time of this study, the DrugBank database contained 7000 approved, withdrawn, investigational, and experimental drug compounds for download [47]. Due to limited experimental data for model validation, we developed and applied a three-tiered docking protocol to predict potential HLA-B*57:01 liable compounds from DrugBank. First, docking was performed using peptide P1 (PDB: 3VRI) to identify all the P1 active compounds, then the P1 actives were screened against peptide P2 (PDB: 3VRJ), and finally, the P1 and P2 actives were screened against peptide P3 (PDB: 3UPR). Using this novel screening protocol, we identified several potentially HLA-B*57:01 liable compounds that have a highly similar binding mode with abacavir and shared activity for three co-binding peptides; thus, increasing the probability of our model to identify true HLA-B*57:01 binders. Overall, this novel virtual screening approach resembles a ‘consensus-like’ modeling workflow which has proven to be highly successful, as demonstrated by Ban et al. in the development of new androgen receptor inhibitors [48]. The development of reliable and inexpensive in silico models for the prediction of HLA-mediated ADRs is key for patient safety and the advancement of Precision Medicine. This new study attempts to demonstrate the usefulness of a novel molecular docking workflow for identifying HLA-B*57:01 liable compounds from the whole DrugBank database.

## Methods

### Preprocessing of the DrugBank database

The compounds we used for our virtual screening targeting the HLA-B*57:01 variant were extracted from the DrugBank database (https://www.drugbank.ca/). DrugBank is a readily available online database that to-date contains well over 8000 entries including FDA approved small molecule drugs, FDA approved biotech (protein/peptide) drugs, withdrawn drugs, and experimental drugs [47]. At the time of our study, there were only 7097 compounds available for download. When performing any virtual screening analysis on such a large dataset, it is essential to ensure that the structural data has been thoroughly curated to avoid erroneous predictions [49–51]. After downloading the DrugBank database, we used the Knime Analytics Platform [52] to conduct data curation using the RDKit Normalization node [53]. The RDKit normalization node verifies the chemical correctness of imported structures by removing bad molecules, identifying fragments, removing unclear bond assignments, identifying erroneous and ambiguous stereo assignments and identifying atom clashes [53]. After normalization of the DrugBank database, we used ISIDA Duplicates to identify and remove duplicate compounds from the file [54]. This curated file was then further pre-processed using LigPrep from the Schrodinger Suite to generate the 3D coordinates of all the curated compounds in addition to exact protonation and tautomeric states at biological relevant pH (pH = 7 ± 2) [55, 56].

### Virtual screening of DrugBank by 3D molecular docking

In our previous study [44], we conducted an in-depth analysis of the capabilities of structure-based molecular docking as a reliable prediction tool for detecting HLA-B*57:01 liable compounds. Herein, using the three curated protein structures (PDB: 3VRI, 3VRJ, and 3UPR) [15, 16], we integrated and applied our models into one consensus docking protocol towards the screening of the whole DrugBank database. Briefly, the protein structures were curated using the Schrodinger Suite’s Protein Preparation Wizard [55, 57] where missing side chains were generated using PRIME [58–60], tautomeric states generated with EPIK [61–63], and an overall energy minimization was performed with the OPLS3 force field [64]. Previously, we thoroughly investigated the binding environment for each X-ray crystal 3VRI, 3VRJ, and 3UPR and discovered that the co-binding peptide had a significant impact on a drug’s binding ability [44]. Furthermore, the co-binding peptides had very distinct amino acid sequences. The peptide from crystal 3VRI will be referred to as P1 (sequence: RVAQLEQVYI), the peptide from crystal 3VRJ will be referred to as P2 (LTTKLTNTNI), and the peptide from crystal 3UPR will be referred to as P3 (HSITYLLPV).

**Fig. 1:**
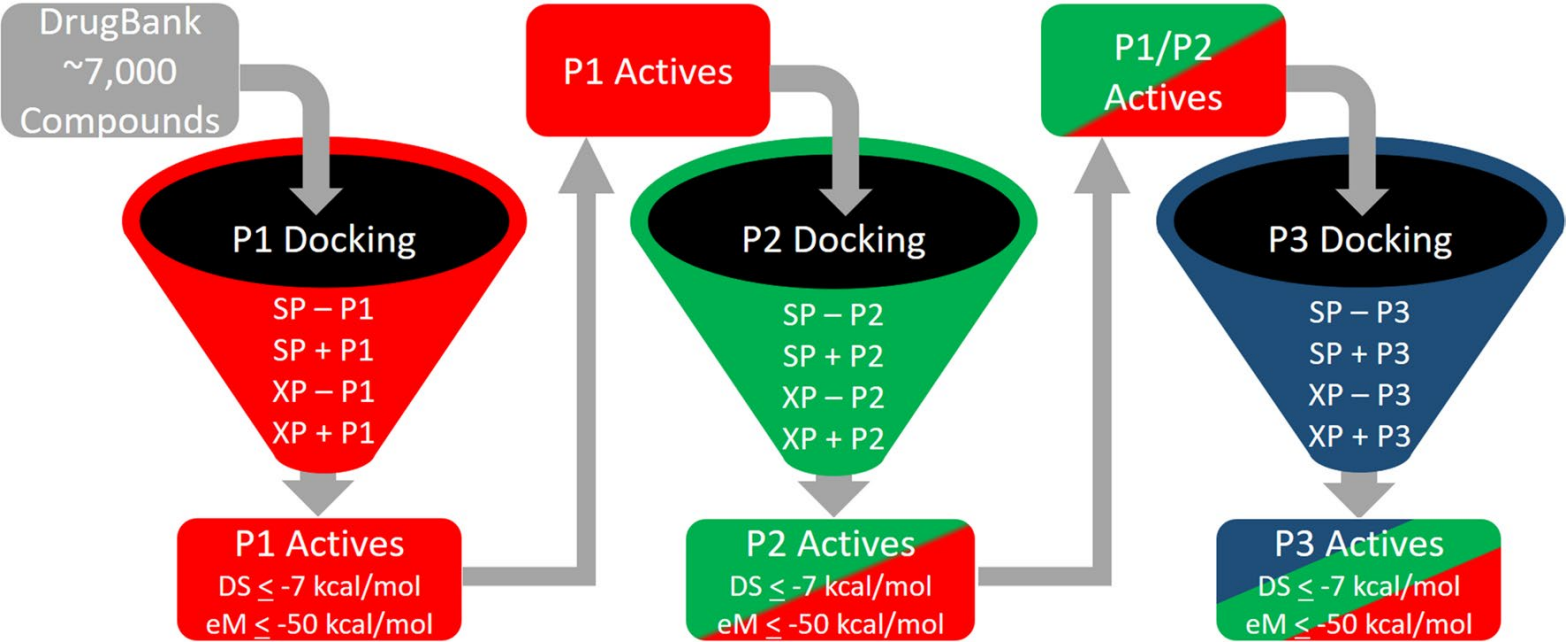
Schematic of virtual screening protocol used to molecular dock DrugBank

Our molecular docking platform for screening a drug’s ability to bind the HLA-B*57:01 variant was built upon our peptide-specific docking models using the three X-ray crystals 3VRI, 3VRJ, and 3UPR. The docking workflow is illustrated in Fig. 1. Molecular docking was conducted using GLIDE from the Schrodinger Suite and compounds were scored using both SP and XP scoring functions [65–68]. This consensus docking was conducted in the presence and absence of peptide using both SP and XP scoring functions [44]. Selected “active” compounds were determined using empirical thresholds for their associated Docking Score (DS) and eModel Score (eM), where any active compound had a DS ≤ −7 kcal/mol and an eM ≤ −50 kcal/mol [44, 69, 70]. The reliability and variance for measured DS has been well-documented by Friesner et al. [68], but the variance for measured eM scores is unavailable because this parameter is strictly a theoretical measure of a ligand’s conformational stability. Though eM was used to determine if a compound was active, this scoring threshold was not used for direct comparison between compounds. As shown in Fig. 1, tier 1 of our docking protocol consisted of docking compounds with crystal 3VRI and peptide P1; first, docking was conducted using the SP scoring function *without* peptide P1. Then after removing predicted non-binders (or inactives), docking of the remaining compounds was performed using the SP scoring function *with* peptide P1. Once more predicted non-binders were removed and the above procedure was repeated with the XP scoring function (XP – P1 and XP + P1). Prior to any round of docking, it is important to underline that duplicate compounds were removed to avoid introducing a conformational bias into our model and the remaining compounds were re-optimized as described in “Preprocessing of the DrugBank database”. After the docking with X-ray crystal 3VRI was completed, the predicted binders (or active compounds) with peptide P1 were docked using the 3VRJ crystal in the presence and absence of peptide P2 (tier 2). The same approach used with 3VRI and P1 was used for docking with 3VRJ and P2. Finally, all the P1 and P2 predicted “active” drugs were docked with 3UPR and P3 (tier 3). This consensus docking protocol produced a refined dataset of drugs with predicted binding modes for peptides P1, P2, and P3 with DS and eM scores that matched all our docking thresholds for determining a compounds’ binding potency towards HLA-B*57:01.

### Analysis of predicted DrugBank HLA-B*57:01 liable compounds

After completing the consensus molecular docking using peptides P1, P2, and P3, the chemical space of predicted DrugBank HLA-B*57:01 liable compounds was explored. First, in order to understand the 2D-structural similarity of predicted actives with the known active abacavir, the MACCS fingerprints of all active compounds were computed [71]. Then, the pairwise Tanimoto similarity score was computed using the MACCS 166-bit fingerprint (T_2D_) with abacavir as the reference compound [72]. The Tanimoto similarity score was computed using Eq. 1,

1 $${\text{T}}_{\text{c}} = \frac{{{\text{b}}_{\text{c}} }}{{{\text{b}}_{1} + {\text{b}}_{2} \, *\,{\text{b}}_{\text{c}} }}$$

where T_c_ is the Tanimoto similarity, b represents the number of computed bits that are shared by both compounds (b_c_), unique to molecule 1 (b_1_), and unique to molecule 2 (b_2_) [72].

However, because our docking workflow specifically identified compounds that were predicted to be HLA binders in presence of all three peptides P1, P2, and P3, we wanted to specifically examine and analyze the binding modes for those hit compounds. It has been reported that interaction fingerprints are appropriate to evaluate molecular docking performance due to their accurate representation of docking poses [73]. As such, the binding environment was analyzed by computing the 3D protein–ligand interaction fingerprints (T_IF_) between each drug and the amino acids of the antigen-binding pocket of HLA-B*57:01 [74, 75]. These fingerprints notably take into account H-bond donor and -acceptor interactions, π–π stacking, electrostatics, and hydrophobic interactions [74, 75]. Next, hierarchical clustering was performed, where the distance matrix between drugs was measured using the Jaccard Distance Matrix as implemented in the R package *vegan* [76]. Then, the Ward Linkage [77] was used to measure the distance between groups as implemented in the R package *gplots* [78]. Finally, the binding modes of the hit compounds were inspected manually.

### Comparison to Metushi et al. model

The study by Metushi et al. [42] identified seven compounds from their in silico analysis that we prepared for docking using LigPrep and EPIK. These compounds were docked using SP and XP scoring functions with peptides P1, P2, and P3 for direct comparisons with our model. Additionally, a recently published X-ray crystal structure (PDB: 5U98) from Yerly et al. [19] has identified a fourth peptide, P4 (VTTDIQVKV), that can bind with HLA-B*57:01 in the presence of abacavir. Notably, both peptides P3 and P4 were incorporated into peptide binding affinity assays for HLA-B*57:01 in the presence of acyclovir [42].

After docking all of the Metushi et al. compounds in our model (and with peptide P4) we conducted molecular dynamic simulations to explore the stability of docked acyclovir with peptide P3. Additionally, molecular dynamic simulations were performed with abacavir and peptide P3 for a baseline comparison. Future molecular dynamic simulations with additional peptides and drug combinations are currently underway and will be discussed in a later publication. All molecular dynamic simulations were performed using Desmond as implemented in the Schrödinger Suite [79–81]. Systems were prepared in 10 × 10 × 10 Å buffered cubic box with a TIP3P solvent model. NPT simulations at 300 K were then performed with an OPLS3 force field [64, 81–83] for 20 ns with a recording interval of 1 ps for both trajectory and energy calculation. Prior to each simulation, Desmond’s default relaxation protocol was performed to equilibrate the system of interest [79–81]. Molecular dynamic trajectories were then analyzed for protein, peptide, and ligand RMSDs and protein–ligand interactions using the Schrödinger suite.

## Results and discussion

### Data curation and molecular docking workflow

This study was conducted using > 7000 approved and experimental drugs available in the DrugBank database [47]. Due to the high impact that non-standardized structural data can have upon a model’s predictive reliability and overall reproducibility, our first task was to clean and standardize the DrugBank dataset [49–51] as described in the “Methods” section. This resulted in a curated dataset of exactly 6094 compounds that were used for molecular docking targeting the HLA-B*57:01 variant. After generating biologically relevant protonation (pH = 7 ± 2) and tautomeric states using LigPrep and EPIK [55, 56, 61, 62], we obtained a total of 20,097 initial poses for docking at the HLA-B*57:01 variant. Again, molecular docking was performed using our new three-tiered workflow (where each tier represents the X-ray crystals 3VRI, 3VRJ, and 3UPR) relying on GLIDE and both SP and XP scoring functions from the Schrodinger Suite as described in “Virtual screening of DrugBank by 3D molecular docking” and shown in Fig. 1 [65–68]. Docked drugs were considered to be HLA-B*57:01 binders (or “active”) if the docked pose had a measured DS ≤ −7 kcal/mol and an eM ≤ −50 kcal/mol [44, 69, 70, 84].

**Fig. 2:**
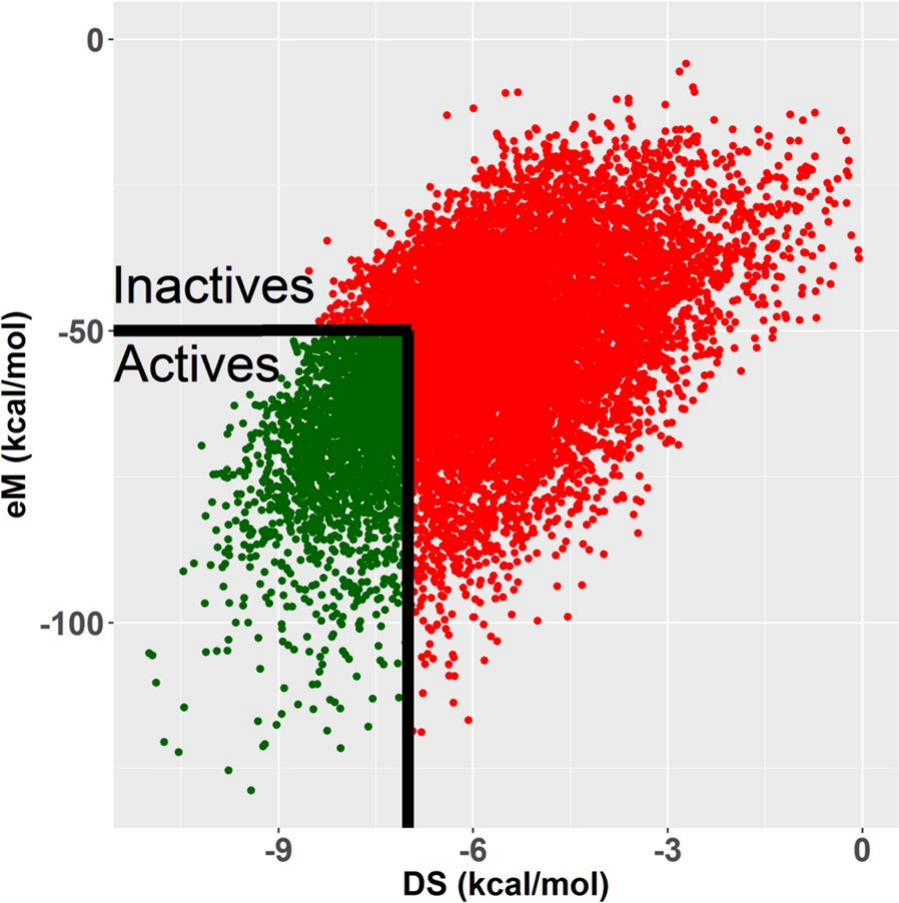
Screening of docked compounds to identify actives (DS ≤ −7 kcal/mol and eM ≤ −50 kcal/mol). Data shown is from SP − P1 round of docking for 15,044 binding conformations

First, molecular docking was performed using the 3VRI crystal in the absence of P1 using the SP scoring function (SP − P1). Initially, out of the 20,097 drug conformations considered for docking, only 15,044 entries were successfully docked using SP − P1 parameters. After applying our active selection thresholds (DS ≤ −7 and eM ≤ −50 kcal/mol), there were only 2931 conformations that remained. Next, duplicates were removed from the data set which resulted in 2072 unique hit compounds under the SP − P1 condition (see Fig. 2). Once duplicates were removed, the SP − P1 active compounds were once more subjected to LigPrep and EPIK optimization before being used in the SP + P1 round of docking. The removal of duplicates after each round of docking was performed to avoid docking of duplicate conformations. One assumption we wanted to avoid in our docking protocol was that the same conformation of a drug would be the same ‘active’ conformation in the presence of peptides P1, P2, or P3. Our previous study had shown that some drugs (e.g., pazopanib) would adopt different binding conformations in the presence or absence of a co-binding peptide [44]. Attempting to avoid this bias also required repetitive rounds of LigPrep and EPIK optimization steps to ensure that selected active compounds comprised all their relevant tautomeric and conformation states prior to the next step of docking.

Our docking protocol from tier 1 using crystal 3VRI and peptide P1, as shown in Fig. 1, identified 619 HLA-B*57:01 liable compounds using both SP and XP scoring functions when peptide P1 is the specific co-binding peptide. The second round of docking was performed using crystal 3VRJ which contained the co-binding peptide P2. Following the same sequential docking procedure (SP − P2, SP + P2, XP − P2, and XP + P2), we identified 75 drugs that passed our thresholds for both co-binding peptides P1 and P2 (Fig. 1). The final stage of our consensus molecular docking used these 75 P1/P2 active drugs and docked them using crystal 3UPR with co-binding peptide P3 (SP − P3, SP + P3, XP − P3, and XP + P3, see Fig. 1). This last round of docking ultimately identified a rather small set of 22 approved, experimental or investigational drugs from DrugBank that passed all our docking thresholds in the presence and absence of peptides P1, P2, and P3.

The ideal docking study would have conducted complete and independent full screens of all DrugBank compounds towards all three crystals 3VRI, 3VRJ, and 3UPR without any removal of compounds until all docking scenarios would have been completed. However, this approach was determined to be computationally expensive (especially with the XP scoring function) and is believed to have resulted in a very similar outcome as our consensus docking protocol was reasonably strict (if only predicted active drugs at all three peptides were selected). Furthermore, only drugs that were forecasted as binders in the presence of all three peptides would be considered as ‘active’ because these compounds would most closely resemble the binding mode of abacavir in HLA-B*57:01 and our model’s applicability domain (still being abacavir-specific) [44].

### Analysis of 22 predicted HLA-B*57:01 liable DrugBank compounds

**Fig. 3:**
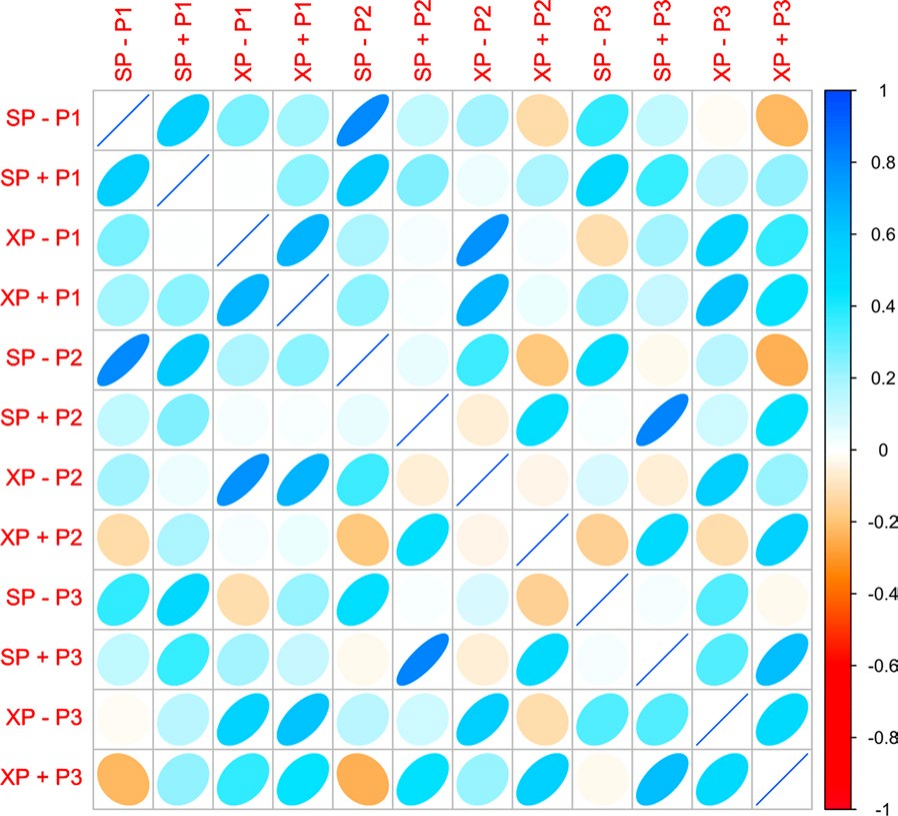
Pearson correlation matrix between active compounds from molecular docking filters (Plot generated using R with CorrPlot (ellipse method))

Twenty-two potential HLA-B*57:01 binders were identified using the molecular docking protocol. First, we plotted the Docking Scores (DS) of these compounds and analyzed their variations based on the type of co-binding peptides. The Pearson correlation coefficients [85] between all Docking Scores are given in Fig. 3; a similar analysis was also done for eM scores (see Additional file 1: Figure 1). Interestingly, when either the SP or XP scoring function was used with peptide P1, there was a reasonable correlation (R > 0.65) when the same scoring functions were used with peptides P2 or P3; however, when the SP scoring function was used for peptides P2 and P3, the observed Pearson correlation was greater than 0.8. For example, the Pearson correlation coefficient was approximately zero between XP + P1 and XP + P2 DS results and was 0.4 when measured between the XP + P1 and XP + P3 results (Fig. 3). The best correlation was observed between the SP + P2 and SP + P3 DS results with a measured Pearson coefficient of 0.8 (Fig. 3). There was a high degree of observed correlations between docking conditions for measured eM scores (Additional file 1: Figure 1).

The DrugBank database includes chemicals classified under various categories [47]: approved, investigational, illicit, and experimental. The docking platform identified 22 active compounds with two drugs being fully approved (*Roflumilast* and *Ramosetron*), two drugs that were flagged as approved and investigational (*Clofarabine* and *Nelarabine*), one drug that was flagged as approved, investigational, and illicit (*Zaleplon*), two drugs that were solely investigational (*Isatoribine* and *Tecadenson*), and 15 drugs that are considered experimental. Table 1 provides information regarding those 22 hit drugs from our screening sorted by their DrugBank IDs along with their generic and/or IUPAC name and T_2D_ similarity coefficient towards abacavir.

**Table 1:** Twenty-two predicted HLA-B*57:01 drugs from DrugBank (with abacavir, DB01048) and measured T_2D_ Similarity scores using abacavir as the reference compound

DATABASE_ID	GENERIC_NAME	Class	T_2D_
DB00631	Clofarabine	Approved; investigational	0.62
DB00962	Zaleplon	Approved; illicit; investigational	0.49
DB01048	Abacavir	Approved; investigational	1.00
DB01280	Nelarabine	Approved; investigational	0.61
DB01656	Roflumilast	Approved	0.35
DB09290	Ramosetron	Approved	0.48
DB04860	Isatoribine	Investigational	0.55
DB04954	Tecadenoson	Investigational	0.61
DB01959	3,5-Dimethyl-1-(3-nitrophenyl)-1h-pyrazole-4-carboxylic acid ethyl ester	Experimental	0.36
DB02096	FR221647	Experimental	0.60
DB02407	6-*O*-cyclohexylmethyl guanine	Experimental	0.58
DB02502	8-hydroxy-2′-deoxyguanosine	Experimental	0.60
DB02984	4-[3-Methylsulfanylanilino]-6,7-dimethoxyquinazoline	Experimental	0.35
DB03365	4-[3-Hydroxyanilino]-6,7-dimethoxyquinazoline	Experimental	0.42
DB03749	4-(1h-imidazol-4-yl)-3-(5-ethyl-2,4-dihydroxy-phenyl)-1h-pyrazole	Experimental	0.40
DB03807	1-(2-Chlorophenyl)-3,5-dimethyl-1h-pyrazole-4-carboxylic acid ethyl ester	Experimental	0.40
DB04518	3-[4-(2,4-Dimethyl-thiazol-5-yl)-pyrimidin-2-ylamino]-phenol	Experimental	0.46
DB04769	5-Quinoxalin-6-ylmethylene-thiazolidine-2,4-dione	Experimental	0.35
DB07051	3,5-Dimethyl-1-phenyl-1*H*-pyrazole-4-carboxylic acid ethyl ester	Experimental	0.43
DB07151	4-(4-Hydroxy-3-methylphenyl)-6-phenylpyrimidin-2(5*H*)-one	Experimental	0.32
DB08048	4-(6-Hydroxy-1*H*-indazol-3-yl)benzene-1,3-diol	Experimental	0.37
DB08485	(1*S*,4*S*,5*S*)-1,4,5-trihydroxy-3-[3-(phenylthio) phenyl]cyclohex-2-ene-1-carboxylic acid	Experimental	0.19

Overall, our screening protocol interestingly identified potential HLA-B*57:01 compounds with a low degree of similarity with abacavir. To examine this structural dissimilarity, we built a similarity matrix based on compounds’ MACCS keys and using abacavir as the active reference probe (Table 1). When the pairwise Tanimoto similarity score (the closer to 1, the most similar) was calculated between abacavir and all the 22 hits, we observed the resulting 2D similarity coefficients ranging from 0.18 to 0.62. The least similar compound was DB02984, an experimental drug; whereas the most similar compound was DB00631 (*clofarabine*), an approved anti-cancer agent.

**Fig. 4:**
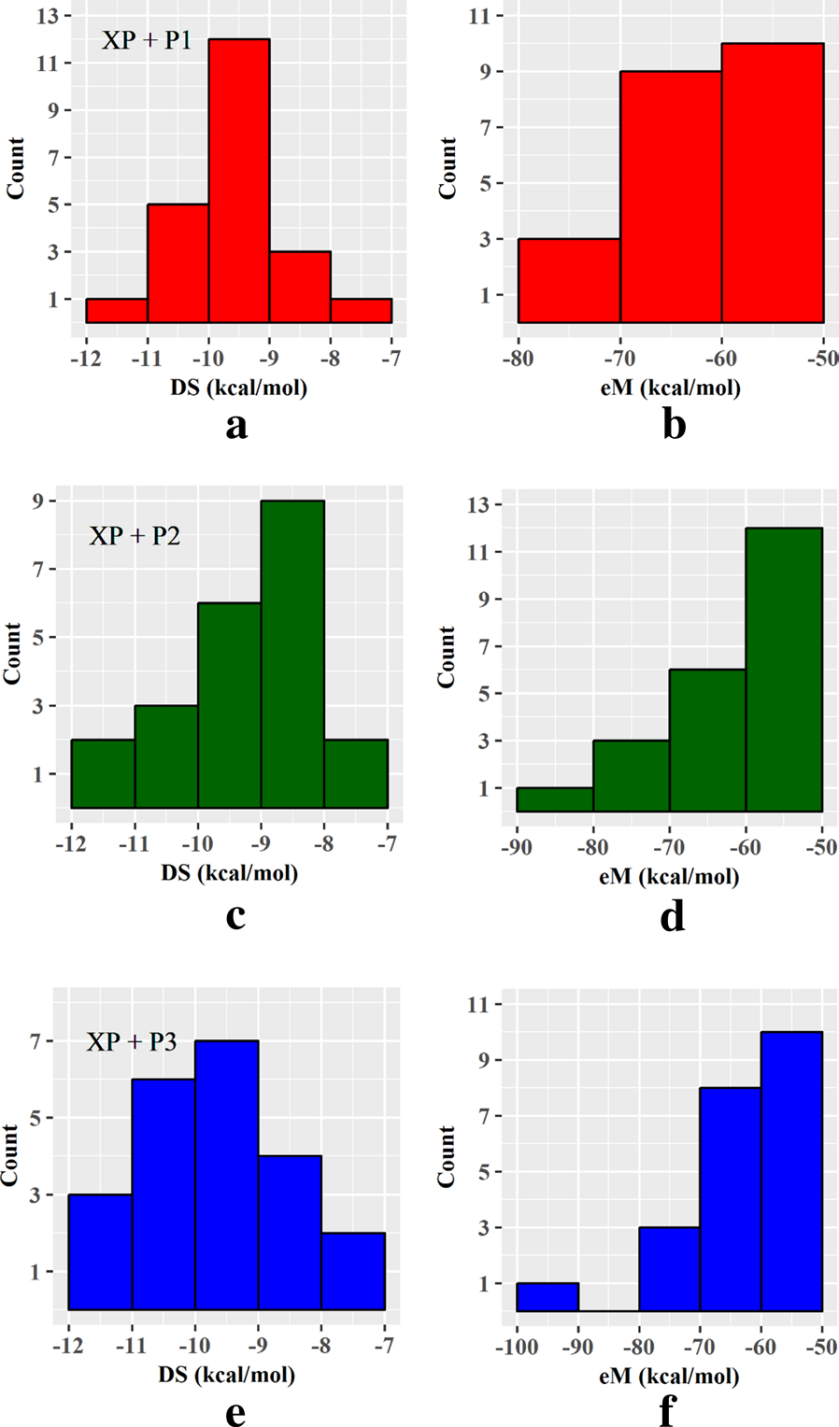
DS and eM distributions for the 22 active compounds using the XP + Pn condition (where n is equal to 1, 2, or 3 depending on the peptide utilized in docking). **a** Distribution of XP + P1 DS, **b** distribution of XP + P1 eM scores, **c** distribution of XP + P2 DS, **d** distribution of XP + P2 eM scores, **e** distribution of XP + P3 DS, **f** distribution of XP + P3 eM scores

Next, the DS and eM distributions of the 22 predicted drugs were analyzed for peptides P1, P2, and P3. These distributions using the XP + P_*n*_ condition, where n is equal to 1, 2, or 3 respective to the co-binding peptide, are provided in Fig. 4. Interestingly, there are four drugs affording DS between − 9 and − 7 kcal/mol, 12 drugs with DS between − 10 and − 9 kcal/mol, five drugs with DS between − 11 and − 10 kcal/mol, and only one drug reaching a DS between − 12 and − 11 kcal/mol (DB08485) as shown in Fig. 4a. The eM distributions were more conserved as 10 of the drugs had eM values ranging from − 60 to − 50 kcal/mol, only 9 drugs were found in the range of − 70 to − 60 kcal/mol, and three drugs had eM scores within the range of − 80 to − 70 kcal/mol (Fig. 4b). When P2 was employed for docking, half of the drugs (11 out of 22) were observed with a DS ranging from − 9 to − 7 kcal/mol, six drugs had DS between − 10 and − 9 kcal/mol, three drugs had DS between − 11 and − 10 kcal/mol, and two drugs (DB04954 and DB07151) had DS between − 12 and − 11 kcal/mol (Fig. 4c). Twelve drugs afforded eM scores ranging from − 60 to − 50 kcal/mol, six drugs were observed with eM scores between − 70 and − 60 kcal/mol, three drugs with eM scores between − 80 and − 70 kcal/mol, and one drug (DB01048) with an eM score between − 90 and − 80 kcal/mol (Fig. 4d). These distributions resemble those observed for peptide P1, even though there are slightly less compounds affording the lowest DS and eM scores. Interestingly, the distributions were significantly altered when docking with peptide P3. There were six drugs with DS between − 9 and − 7 kcal/mol, seven drugs with DS between − 10 and − 9 kcal/mol, six drugs with DS between − 11 and − 10 kcal/mol, and three drugs (DB04860, DB07151, and DB08485) with DS between − 12 and − 11 kcal/mol (Fig. 4e). There were 18 drugs with a measured eM score between − 70 and − 50 kcal/mol, three drugs with eM scores between − 80 and − 70 kcal/mol, and one drug (DB01048) with an eM score between − 100 and − 90 (Fig. 4g). All XP + Pn DS values are provided in Table 2 and DS and eM scores under all conditions are available in Additional file 1: Tables 1 and 2, respectively.

**Table 2:** Docking Scores (DS) of 22 active compounds identified from screening of DrugBank

DRUGBANK ID	DS XP + P1	DS XP + P2	DS XP + P3
DB00631	− 8.06	− 8.61	− 7.98
DB00962	− 9.46	− 8.02	− 9.14
DB01048	− 9.60	− 9.20	− 10.06
DB01280	− 9.29	− 8.86	− 10.18
DB01656	− 9.79	− 9.74	− 9.45
DB09290	− 10.20	− 8.44	− 8.84
DB04860	− 9.42	− *10.47*	− *11.22*
DB04954	− 9.65	− *11.05*	− 10.54
DB01959	− 9.02	− 8.62	− 9.14
DB02096	− 9.90	− 9.21	− 9.10
DB02407	− 9.20	− 7.36	− 7.79
DB02502	− 9.81	− 8.99	− *10.29*
DB02984	− *10.60*	− 8.26	− 8.20
DB03365	− 9.81	− *10.00*	− 10.17
DB03749	− 8.61	− 9.01	− 8.87
DB03807	− *10.52*	− 8.53	− 9.73
DB04518	− *10.40*	− 9.32	− 9.63
DB04769	− 8.87	− *10.10*	− *10.22*
DB07051	− 9.69	− 9.23	− 9.28
DB07151	− *10.58*	− *11.04*	− *11.25*
DB08048	− 7.75	− 8.32	− 8.79
DB08485	− *11.25*	− 7.57	− *11.51*

The top-5 binders for each docking condition (XP + P1, XP + P2, and XP + P3) are in italics

### Hierarchical clustering of the top-22 predicted HLA-B*57:01 liable DrugBank compounds

After docking was completed, the binding modes of the 22 predicted HLA-B*57:01 liable DrugBank compounds were analyzed using interaction fingerprints due to their accurate representation of docking poses [73]. The respective binding modes of proposed active drugs were analyzed using 3D protein–ligand interaction fingerprints, which map out the intermolecular interactions between the ligand and protein binding pocket [74, 75]. This type of fingerprint can be further used to generate an interaction fingerprint Tanimoto (T_IF_) similarity coefficient by comparing the interaction fingerprints of the 22 predicted active drugs versus that of the native binding mode of abacavir.

**Fig. 5:**
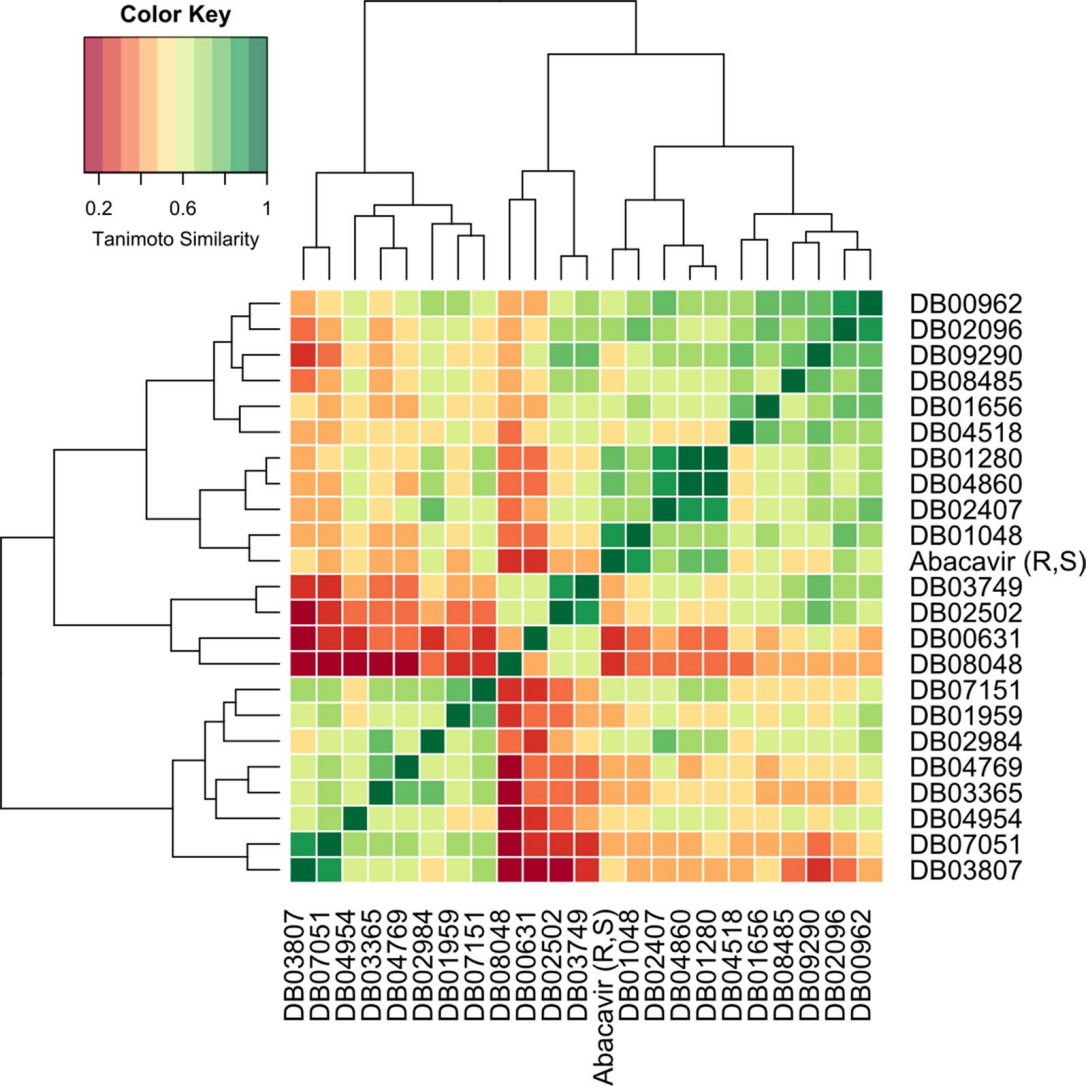
Drug binding mode fingerprint similarity matrix clustered using the Ward algorithm. Red indicates a low tanimoto similarity (0–0.3), yellow indicates moderate tanimoto similarity (0.3–0.7), and green indicates high tanimoto similarity (0.7–1.0)

Once the interaction fingerprints were generated for all 22 predicted HLA-B*57:01 compounds, we conducted a hierarchical clustering using those fingerprints as input descriptors; distances between compounds were measured with a Jaccard distance index as implemented by the *vegan* package in R [76] and distances between clusters were measured using a Ward linkage as implemented by the *gplots* package in R [77, 78]. The hierarchical clustering results using the binding modes from XP + P1 docking are provided in Fig. 5. There were six observed clusters of compounds on the dendrogram. Cluster 1 contained two compounds (DB03807 and DB07051), Cluster 2 consisted of six compounds (DB04954, DB003365, DB04769, DB02984, DB01959, and DB07151), Cluster 3 had two compounds (DB08048 and DB00631), Cluster 4 also had two compounds (DB02502 and DB03749), Cluster 5 included four DrugBank compounds and native abacavir (native abacavir, DB01048, DB02407, DB04860, and DB01280), and Cluster 6 had six compounds (DB041518, DB01656, DB09290, DB02096, and DB00962).

Interestingly, there were four DrugBank compounds clustered with native Abacavir in Cluster 5 (DB01048, DB02407, DB04860, and DB01280). DB01048 is the actual DrugBank ID for abacavir. This indicates that from a database of 7000 compounds, our docking platform could successfully re-identify this drug as HLA-B*57:01 binder. However, T_IF_ between the two binding modes is not exactly 1.0 because the hydroxyl group of abacavir from DrugBank is not H-bonding with TYR74 (like the actual native abacavir); furthermore, the measured RMSD between native abacavir and DB01048 is 1.11 Å, which occurs due to differing orientations of the cyclo-pent-2-en-yl-methanol functional groups. This conformational difference is a result of the flexible binding mode of abacavir. Previously, we reported that the hydroxyl group could H-bond with the ALA3 backbone of peptide P1 [44]. Clearly, molecular dynamic simulations are needed to further investigate the preferred binding orientation of the hydroxyl group of abacavir. The closest cluster to Cluster 5 was Cluster 6, which contained six compounds that had T_IF_ ranging from 0.5 to 0.7; the furthest cluster from Cluster 5 was Cluster 1, which contained two compounds with T_IF_ less than 0.5 (Fig. 5). Notably, Clusters 1–4 had low measured T_IF_ values when compared to the binding mode of native abacavir.

Unexpectedly, when hierarchical clustering was conducted using the interaction fingerprints from peptides P2 and P3, the same drugs were not clustered together (Additional file 1: Figures 2 and 3). Clustering with peptide P2 revealed that only abacavir and DB01048 (DrugBank abacavir) were clustered together (Additional file 1: Figure 2); P3 clustering resulted in the drugs DB00962, DB04954, and DB01048 all clustering with abacavir (Additional file 1: Figure 3). Clearly, these results demonstrated again that the co-binding peptide is extremely important in a drug’s ability to bind with HLA-B*57:01. The binding modes of the clustered drugs from XP + P1 screening were then selected for further analysis and comparison with the XP + P2 and XP + P3 screening results.

**Fig. 6:**
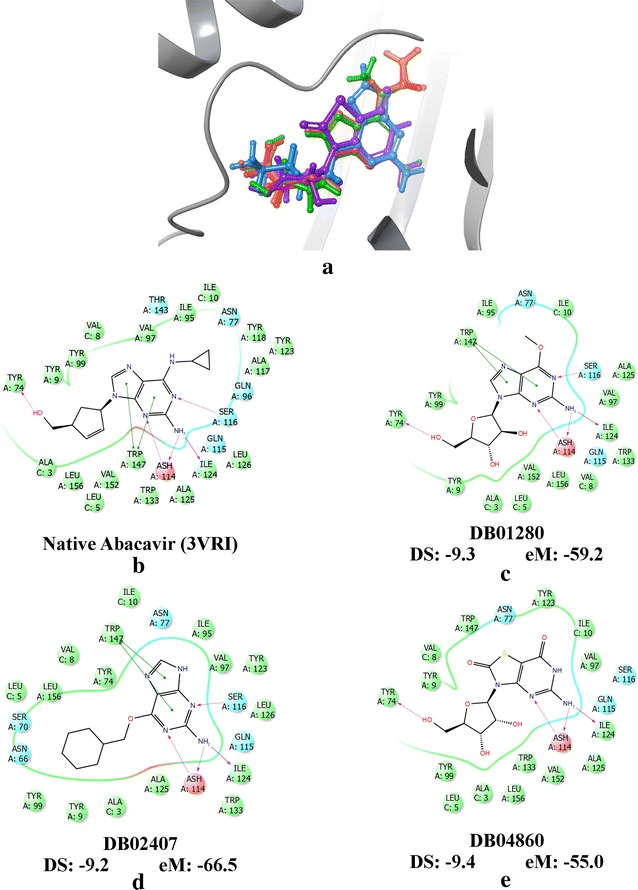
Three compounds determined most likely to be active from Ward clustering using interaction fingerprint (DB01280, DB02407, and DB04860). **a** Superimposition of clustered drugs, abacavir (red), DB01048 (abacavir from DrugBank, orange), DB01280 (purple), DB02407 (green), and DB04860 (blue). Binding modes of **b** native abacavir (PDB: 3VRI), **c** DB01280, **d** DB02407, and **e** DB04860 with the measured DS and eM scores from XP + P1 docking. The binding mode of DB01048 is not shown

The compounds from Cluster 5 (abacavir (*native*), DB01048, DB01280, DB02407, and DB04860) were superimposed (Fig. 6a) in the binding pocket of HLA-B*57:01 and their respective protein–ligand interactions were analyzed (Fig. 6b–e). The same set of drugs was superimposed in the HLA-B*57:01 binding pocket from XP + P2 (Additional file 1: Figure 4A) and XP + P3 (Additional file 1: Figure 5A) screening. Additionally, the binding modes of these same drugs were analyzed with peptides P2 and P3 (Additional file 1: Figures 4B-E and 5B-E), respectively.

The 3D superimposition revealed that these top drugs occupy similar binding domains as abacavir in the HLA-B*57:01 binding pocket. Interestingly, the three top performing drugs share a significant number of structural similarities with native bound abacavir from X-ray crystal 3VRI. Notably, two of the clustered drugs (DB01280 and DB02407) share the same purine scaffold as abacavir with key substitutions occurring at the six and nine positions of the purine ring. The six position of abacavir has a cyclopropylamino functional group, while the nine position has a cyclopent-2-en-yl-methanol functional group. These differing functional groups have a significant impact upon the observed binding modes of each drug in the pocket. For example, the methanol substituent of abacavir provides H-bonding with TYR74, while the purine scaffolding provides several H-bonds with ASH114 (neutral ASP), SER116, and ILE124; additionally, the purine scaffold provides stabilization via π–π stacking with TRP147 (Fig. 6b). These same AA interactions are observed in the binding modes of native abacavir with P2 (PDB: 3VRJ) and P3 (PDB: 3UPR), respectively (Additional file 1: Figures 4B and 5B).

Compound DB01280 (*nelarabine*) shares the same AA interactions as abacavir, but has key substitutions at the six and nine positions of the purine scaffold, see Fig. 6c. DB01280 (*nelarabine*) has a methoxy functional group at the six position and an alcohol functionalized tetrahydrofuran ring (*ribose*) at the nine position. The computed DS for DB01280 (*nelarabine*) was as low as − 9.3 kcal/mol, while the computed eM score was − 59.2 kcal/mol indicating that DB01280 could be predicted to be an HLA-B*57:01 liable compound (Table 2). The binding mode of DB01280 with peptide P2 is similar, but is missing two key H-bonds with ILE124 and TYR74. However, a hydroxyl group from the ribose ring does form an H-bond with the LEU5 peptide backbone of P2 (Additional file 1: Figure 4C). This results in a slightly worst DS of − 8.9 kcal/mol, but an extremely favorable eM of − 75.0 kcal/mol. When DB01280 (*nelarabine*) was docked with P3, the DS was extremely favorable too with a value as low as − 10.2 and eM of − 62.0 kcal/mol. This increased stability most likely results from additional H-bonding of the ribose ring (Additional file 1: Figure 5C). DB01280’s (*nelarabin*e) ribose ring is observed to H-bond with LEU5 of P3, but also has H-bonding with TYR74 occurring with the O-heteroatom of the tetrahydrofuran scaffolding. Interestingly, Cohen et al. reported in 2008 the observance of grade 3 and 4 ADR events resulting in hematologic and neutrophil toxicity during a clinical trial [86]. As DB01280 is a chemotherapy drug used in the treatment of acute T-cell lymphoblastic leukemia, it is likely that this drug’s ADRs are mainly due to its overall cytotoxicity and any association with HLA is unclear at this point.

The experimental drug, DB02407, also has a purine scaffolding like abacavir, but has significant functional group deviations at the six and nine positions. The six position contains a cyclohexylmethoxy functional group which is sterically much larger than abacavir’s cyclopropylamino substituent. Additionally, the nine position of DB02407 is protonated which prevents it from reaching the TYR74 residue; however, the ligand–protein AA interactions surrounding the purine scaffold are conserved between abacavir and DB02407, see Fig. 6d. Even with the missing H-bond, the measured DS and eM scores were extremely favorable for DB02407, − 9.2 and − 66.5 kcal/mol, respectively (Table 2). However, when docking was performed using P2 or P3, the computed DS were less favorable (though still passing our threshold) at − 7.4 and − 7.8 kcal/mol for P2 and P3, respectively. Interestingly, the AA interactions between abacavir and DB02407 are conserved surrounding the purine scaffold for both P2 and P3 (Additional file 1: Figures 4D and 5D). However, the decrease in DS most likely results from the increased steric hindrance from the cyclohexylmethoxy substituent. DB02407 is currently an experimental drug and there is no additional indication provided by DrugBank; as such, no ADR reports are available for this drug.

The last drug identified as a top performer from the (XP + P1) clustering results was the compound DB04860 (*isatoribine*). Instead of a purine scaffolding, DB04860 (*isatoribine*) has an oxoguanine scaffold where the seven position N-heteroatom is a S-heteroatom. A key difference between a purine scaffold and an oxoguanine scaffold is that the six and eight position carbons are fully oxidized carbonyl groups. Additionally, the nine position has an alcohol substituted tetrahydrofuran ring (or ribose) like DB01280 (*nelarabine*). Interestingly, when docking with P1, H-bonds are conserved with ASH114, ILE124, and TYR74, but the H-bond with SER116 is lost due to protonation of the five position N-atom. Additionally, the π–π stacking that was observed with purine scaffolds and TRP147 is no longer present (Fig. 6e). Notably, the loss of π–π stacking does not appear to significantly impact the binding mode stability as the measured XP DS and eM scores were still extremely favorable when docking with P1 at − 9.4 and − 55.0 kcal/mol, respectively (Table 2). Interestingly, when docking with P2 or P3 was performed, the H-bond with ILE124 was no longer observed, but H-bonding was observed between hydroxyl groups of the ribose ring and LEU5 of P2 and TYR5 of P3. Additionally, the O-heteroatom of the tetrahydrofuran substructure of the ribose ring was observed to H-bond with TYR74 when docking with P3 (Additional file 1: Figures 4E and 5E). Overall, DB04860 (*isatoribine*) afforded extremely favorable XP docking results with HLA-B*57:01 when docked with P2 (DS: − 10.5 kcal/mol, eM: − 64.6 kcal/mol) and P3 (DS: − 11.2 kcal/mol, eM: − 53.2 kcal/mol). The drug, DB04860, is an investigational drug used in the treatment of hepatitis C, but was discontinued during clinical trials in 2007 as a prodrug due to overt immunostimulation [87].

Interestingly, the measured T_IF_ similarities from interaction fingerprints compared to native abacavir’s binding mode varied significantly for each peptide as well. When P1 was used, the T_IF_ similarity ranged from 0.2 to 1.0, while both P2 and P3 had the most dissimilar compounds with T_IF_ similarities of 0.4 or greater. These drastic changes in measured T_IF_ likely occur from slight changes in the binding pocket caused by different co-binding peptides P1, P2, and P3. Future studies will attempt to explore this possibility using molecular dynamics and Schrodinger’s peptide docking procedure implemented by GLIDE [88]. All the computed T_IF_ similarity scores derived from drug interaction fingerprints (using native abacavir as the reference compound) are provided in Additional file 1: Table 3 for peptides P1, P2, and P3.

Unexpectedly, when we began looking at the most dissimilar binding modes of DrugBank compounds compared to abacavir’s docking pose, we found that the T_IF_ similarity scores were peptide-dependent. For example, DB00631 (*clofarabine*) was determined to have the least similar binding mode with abacavir for the XP + P1 screening with a T_IF_ similarity score of 0.24 (Additional file 1: Table 3) and a T_2D_ similarity score of 0.62 (Table 1). However, when XP + P2 or XP + P3 screenings were performed, this same compound afforded significantly higher similarity scores of 0.68 and 0.75, respectively. Strangely, when DB00631’s (*clofarabine*) binding mode from XP + P1 was superimposed with the XP + P2 or XP + P3 clofarabine binding modes the measured RMSD was 1.6 and 1.2 Å, respectively; while the superimposition of the XP + P2 and XP + P3 binding modes had a measured RMSD of 0.8 Å. Clearly, the binding conformation of DB00631 was impacted by the co-binding peptide. DB00631 (*clofarabin*e) is an anticancer agent especially used to treat leukemia. Bonate et al. and others reported the observance of several ADRs including febrile neutropaenia and hypotension [89], but these are classical ADRs for such chemotherapy drugs. Unclear is if any HLA-mediated ADR has ever been observed with this drug.

**Fig. 7:**
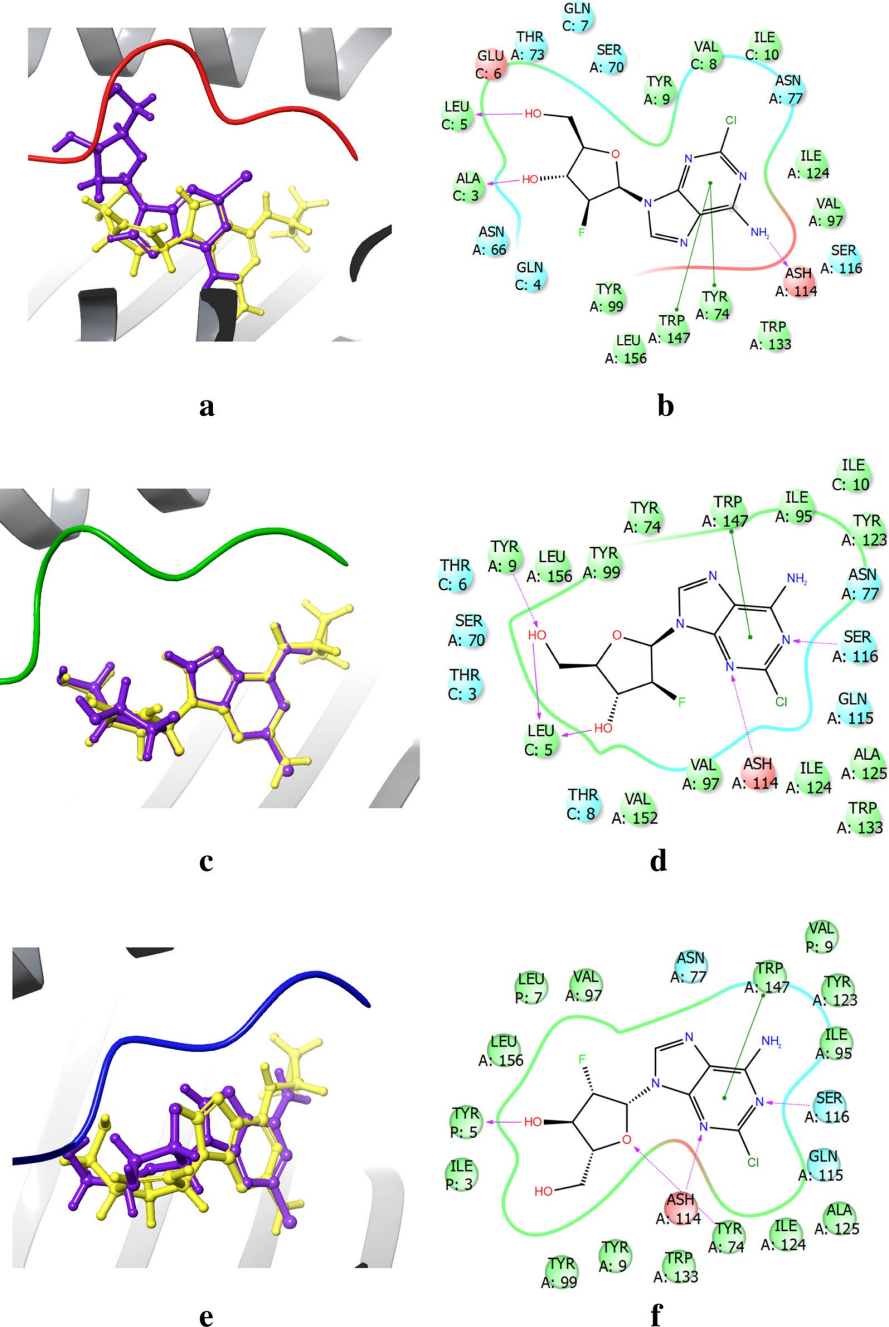
Binding mode analysis of the most dissimilar DrugBank compound, DB00631 (Purple), from abacavir (Yellow) identified using XP + P1 screening. **a** Superimposition of abacavir and DB00631 from XP + P1 screening with P1 shown in red, **b** XP + P1 binding mode of DB00631, **c** Superimposition of abacavir and DB00631 from XP + P2 screening with P2 shown in green, **d** XP + P2 binding mode of DB00631, **e** Superimposition of abacavir and DB00631 from XP + P3 screening with P3 shown in blue, **f** XP + P3 binding mode of DB00631

Next, we superimposed DB00631 (*clofarabine*) with abacavir and analyzed the individual binding modes of clofarabine (Fig. 7). Interestingly, the chemical scaffold of DB00631 shares the same purine subunit as abacavir, but has key differences in functional group placement at the two and six position. Unlike abacavir, the attached amino group is not at the two position, but is instead at the six position; the two position of the purine scaffold, in fact, has a chlorine atom present. Like the compounds DB01280 (*nelarabine*) and DB04860 (*isatoribine*), DB00631 (*clofarabine*) has a ribose like group attached at the nine position of the purine ring, but instead of a hydroxyl group attached to the two position of the tetrahydrofuran ring there is a fluorine atom. These small differences result in significant binding mode differences with peptide P1.

Superimposition between abacavir and DB00631 (*clofarabine*) with P1 revealed that there is minimal overlap between the binding modes of these two drugs (Fig. 7a): only π–π stacking with TRP147 and H-bonding with ASH114 are conserved with the purine scaffold (Fig. 7b). Interestingly, H-bonding with ILE124 is not observed due to the substitution of a chlorine atom at the two position of the purine scaffold. When P2 was incorporated in docking, the superimposition revealed that abacavir and DB00631 occupied similar binding domains as shown in Fig. 7c. Under these conditions, H-bonding with SER116 is regained while there is also H-bonding with LEU5 of P2 (Fig. 7d). Analogous to docking with P2, superimposition of DB00631 and abacavir when docked with P3 revealed a conserved binding mode and H-bond stabilization provided by TYR5 or P3 (Fig. 7e, f). Interestingly, the measured DS were − 8.0 kcal/mol for both P1 and P2, while DS was observed to be − 8.6 kcal/mol. This indicates that even though the binding location of DB00631 fluctuates with different peptides, the overall binding affinity is conserved.

The most dissimilar drug when using interaction fingerprints from the P2 screening was DB04954 (*tecadenoson*) which has a T_IF_ similarity of 0.42. Interestingly, T_IF_ similarity was only slightly increased to 0.56 when docking with peptide P1, but when docked with P3 this drug had a highly similar binding mode as abacavir with a T_IF_ similarity score of 0.81 (these binding modes are not provided). The observed 2D-similarity between the DB04954 (*tecadenoson*) and abacavir scaffolds was 0.61 (Table 1). This compound was observed to have very favorable DS under all docking conditions with DS ranging from − 9 to − 11 kcal/mol (Table 2). DB04954 is an investigational drug used in the treatment of arrhythmia and atrial fibrillation, but has been reported with mild ADR events of headaches, chest pain, and hypesthesia [90, 91]. These mild ADRs are common drug side-effects and it is unclear if these symptoms are caused by a HLA-mediated pathway.

When docking with P3, the most dissimilar binding mode, when compared to abacavir, was DB08048 (an experimental drug) with a T_IF_ similarity of 0.55. However, when docking with P1, the observed T_IF_ was as low as 0.30, whereas the similarity when docking with P2 was 0.48. Interestingly, it was also observed that the T_2D_ similarity score (generated from MACCS fingerprints) between DB08048 and abacavir were also highly dissimilar with a measured value of 0.37 (Table 1). The observed DS scores for DB08048 passed our threshold with measured values of − 7.8, − 8.3, and − 8.8 kcal/mol for peptides P1, P2, and P3, respectively (Table 2). Remarkably, the chemical scaffolding of DB08048 was quite different from abacavir as the purine scaffold was replaced by an indazole scaffold connected to a diol benzene ring. DB08048 is an experimental drug whose primary target listed on DrugBank is the estrogen receptor [47].

Next, using the measured DS scores in Table 2, we determined what the top five strongest HLA-B*57:01 binders were in the presence of P1, P2, and P3. Interestingly, each docking condition resulted in a unique set of top five drugs with some overlaps. The top five binding drugs with P1 were DB02984, DB03807, DB04518, DB07151, and DB08485; which are all classified as experimental drugs. When docking with P2 the top five drugs were DB03365, DB04769, DB04860 (*isatoribine*), DB04954 (*tecadenoson*), and DB0715. Lastly, the top five drugs when docking with P3 provided some overlap with P1 and P2 conditions resulting in the following: DB02502, DB04769, DB04860 (*isatoribine*), DB07151, and DB08485. Notably, all the listed compounds afforded extremely low DS between − 12 and − 10 kcal/mol, which are strong indicators for significant binding affinity. Furthermore, some compounds obtained excellent DS for multiple peptides. The drug DB07151 was a top binder for all three peptides, while DB08485 was a top binder for peptides P1 and P2, and the drugs DB04769 and DB04860 were top binders for peptides P2 and P3.

### Model comparisons to Metushi et al

Herein, we would like to address the recent and excellent study by Metushi et al. [42] who conducted a full in silico to in vitro screening of the ZINC database. In their study, they conducted a 2D-similarity screening of the ZINC database using abacavir as the reference compound. Then taking the most similar compounds from this 2D-screening, Metushi et al. conducted a 3D-similarity screening by superimposing generated 3D conformations with native abacavir and filtered inactive compounds by measured RMSD (with abacavir) [42]. Additionally, compounds that did not share similar structure activity relationships (SAR) as abacavir were also removed [42]. This combination of 2D- and 3D-screening resulted in the identification of 54 compounds that were docked in the HLA-B*57:01 binding site (PDB: 3UPR) using GOLD5.2 and GOLD-Score scoring functions [42]. Based on these docking results, the top seven compounds were selected for in vitro analysis using a previously developed radio-labeled peptide competitive binding assay [92] with three nine mer peptides (M1: KVAKVEPAV, M2: RVAGIHKKV, M3: HSITYLLPV). The seven selected compounds were: Roscovitine (not in DrugBank), cladribine (DB00242), acyclovir (DB00787), arranon (DB01280 or nelarabine), minoxidil (DB00350), sangivamycin (not in DrugBank), and bohemine (not in DrugBank). Notably, Metushi et al. [42] determined that only acyclovir significantly increased peptide binding with HLA-B*57:01 from this radio-labelled peptide competitive binding assay. Acyclovir (DB00787) was then subjected to binding affinity assays with multiple peptides to determine the best HLA-B*57:01-acyclovir-peptide combination for T-cell activation studies. However, it was observed that acyclovir did not induce a T-cell response and was therefore determined to not cause ADR events via a binding mechanism with HLA-B*57:01. Acyclovir is a guanosine analog antiviral used for treatment of herpes zoster (shingles), genital herpes, and chicken pox and has a robust safety profile with limited ADR case reports [42, 93–95].

**Fig. 8:**
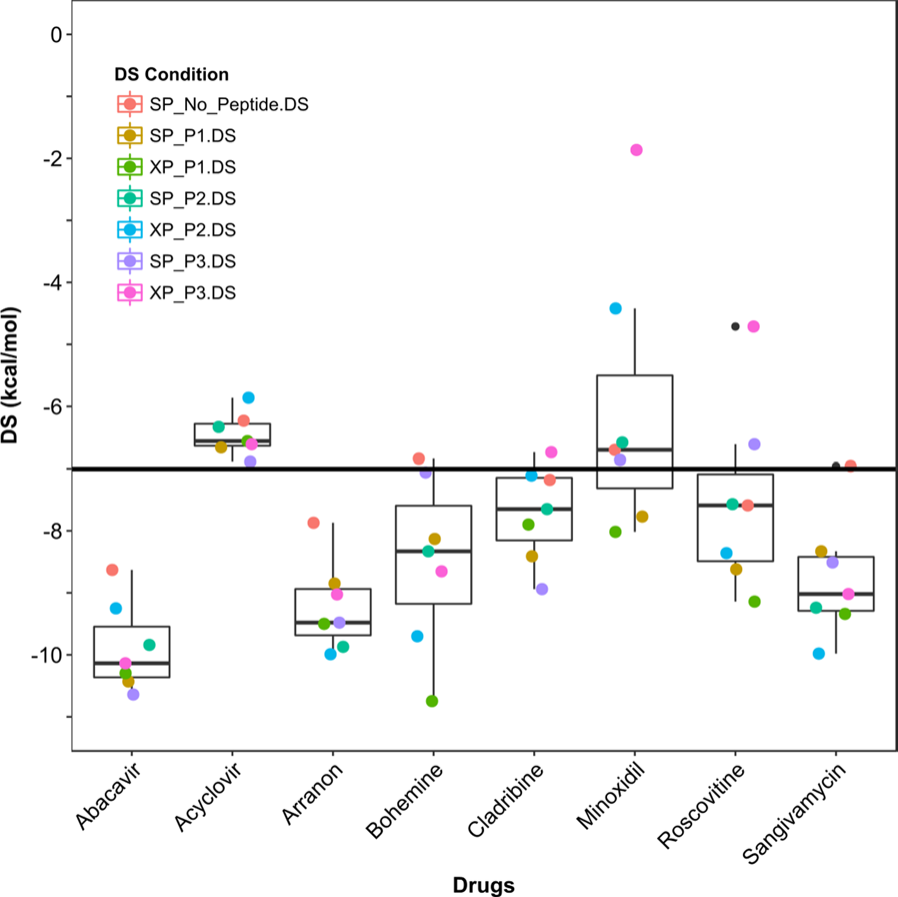
Glide measured DS of abacavir (DB01048) and seven proposed HLA-B*57:01 active compounds proposed by Metushi et al. from the ZINC database. The seven Metushi et al. compounds are: Acyclovir (DB00787), arranon (DB01280 or nelarabine), bohemine, cladribine (DB00242), minoxidil (DB00350), roscovitine, and sangivamycin. Measured DS are reported as boxplots with superimposed 1D-vertical scatter plots with applied horizontal jitter to prevent datapoint overlap. Each data point is color coded per the condition of docking: SP without peptide (salmon), PDB: 3VRI), SP with P1 (gold), XP with P1 (olive green), SP with P2 (green), XP with P2 (turquoise), SP with P3 (light blue), XP with P3 (blue), SP with P4 (purple), and XP with P4 (pink). Peptide P1 corresponds to crystal 3VRI, P2 corresponds to crystal 3VRJ, P3 corresponds to crystal 3UPR, and P4 corresponds to crystal 5U98. The DS threshold (DS ≤ −7 kcal/mol) is marked as a black line on the plot

Interestingly, four of the seven compounds identified by Metushi et al.’s docking procedure [42] can also be found in the DrugBank database (acyclovir, arranon, cladribine, and minoxidil); however, only the compound arranon (DB01280 or nelarabine) was identified as an in silico active compound in both models. Our model identified acyclovir (DB00787), cladribine (DB00242), and minoxidil (DB00350) as inactive compounds that failed at the SP − P1 (PDB: 3VRI), XP − P2 (PDB: 3VRJ), and SP − P1 (PDB: 3VRI) levels of docking, respectively. Notably, as discussed in methods “Virtual screening of DrugBank by 3D molecular docking”, our consensus screening platform discarded inactive compounds after each round of docking to generate a set of “active” compounds with all three peptides P1, P2, and P3. As such, we generated the 3D-conformations of the seven actives proposed by Metushi et al. [42] using LigPrep and docked with peptides P1, P2, and P3 using GLIDE SP and XP scoring functions. Notably, a recent publication by Yerly et al. [19] has solved a fourth X-ray crystal structure of HLA-B*57:01 with bound abacavir and a 9-mer co-binding peptide (PDB: 5U98, P4: VTTDIQVKV). The crystal structure obtained from 5U98 was curated using the same workflow as described in the methods. Since this study does not include experimental validation, we posit that a fourth peptide, P4, allowed a more thorough in silico analysis of the compounds proposed by Metushi et al. Additionally, there are now two peptides that have experimental measured IC_50_ values available for comparison between Metushi et al.’s [42] study and our docking model. This was performed to fully determine why our docking protocol did not identify the same compounds as Metushi et al. The measured DS are provided in Fig. 8 and measured eM scores are provided in Additional file 1: Figure 6.

Using GLIDE docking, it was observed that the only compound identified as active would be arranon (or *nelarabine*, DB01280); all other compounds failed the DS and/or eM thresholds for at least one docking condition. For example, the compound bohemine afforded a DS range of − 10 to − 7 kcal/mol (indicating it is active for DS, Fig. 8), but had multiple conditions with suboptimal eM scores (Additional file 1: Figure 6). Interestingly, the compound cladribine (DB00242) had favorable DS and eM scores for all conditions except when docking with peptide P3 (DS > −7). Acyclovir (DB00787) obtained eM scores that passed our threshold (Additional file 1: Figure 6), but failed the DS threshold under all conditions except when docked with peptide P4 using the SP scoring function (− 8.17 kcal/mol) (Fig. 8).

As noted earlier, Metushi et al.’s study [42] tested in vitro if the seven proposed actives enhanced peptide binding affinity with co-binding peptides M1, M2, and M3, and determined that only the drug acyclovir had a significant impact on binding affinity. Then acyclovir was selected for further evaluation with over 15 different peptides and tested for T-cell activation with an optimized binding peptide. The results from the T-cell activation assay revealed that binding acyclovir did not activate T-cells. Notably, both in silico models used crystal 3UPR (peptide P3 or M3) to conduct virtual screening, but our docking platform also included three additional peptides (P1, P2, and P4) to determine a drug’s binding ability with HLA-B*57:01. Interestingly, Metushi et al. screened both P3 and P4 for their binding affinity with acyclovir. Peptide P3’s binding affinity for HLA-B*57:01 was shown to significantly increase in the presence of acyclovir; an observation that contradicts our model’s prediction. However, Metushi et al. demonstrated that the binding affinity of peptide P4 for HLA-B*57:01 was marginally impacted by acyclovir agreeing with our model’s XP results, but conflicting with our SP results (Fig. 8) [42]. Conflicting results like these demonstrate that molecular docking might not be efficient enough as a stand-alone tool for modeling complex tripartite systems such as HLA-drug-peptide combinations. Furthermore, we want to emphasize that since our screening platform was *not* constructed using a HLA-B*57:01 variant complexed with a T-cell, predicting if a drug binding to HLA-B*57:01 will induce T-cell activation is well beyond the model’s scope and abilities. Our approach might be considered when used to determine if a drug can bind with HLA-B*57:01 when peptides P1, P2, or P3 are present in an abacavir-specific binding mechanism. Clearly, the relationship between HLA-drug binding and T-cell activation needs to be explored in greater detail through a combination of in silico and experimental techniques.

Comparisons between these two very complementary studies can provide valuable insights for the development of future virtual screening workflows for HLA-mediated ADRs (especially for other HLA variants). First, our ensemble docking protocol successfully eliminated six out of the seven proposed compounds by Metushi et al. indicating that incorporating multiple peptides can dramatically improve model efficiency. However, our screening platform did identify arranon (DB01280 or nelarabine) as an active, whereas Metushi et al.’s experimental evidence indicates the opposite; this could be a result of arranon (DB01280 or *nelarabine*) exhibiting peptide specificity for either P1 or P2, but not peptides M1 and M2 that were used in the binding assay. Future experimental validation will likely test this possibility by measuring arranon’s (DB01280) binding affinity towards peptides P1, P2, P3, and P4. The second takeaway from these two studies is that for any structure-based docking model to be successful, multiple co-binding peptides will need to be considered when docking any drug of interest. Clearly, the ideal docking protocol would include all (or a set of most representative) peptides with high affinities for the targeted HLA-variant to ensure experimental success, but in the absence of fully solved HLA-peptide binding modes, this will be a difficult challenge to solve. A recent study by Gürsoy and Smieško [96] tested the reliability of force fields to accurately predict biologically active conformations of drugs, and revealed that conformational accuracy of a force field decreases as the number of rotatable bonds in a compound increases. Obviously, accurately predicting the binding conformation of peptides will be a major obstacle due to the high number of rotatable bonds, despite some significant progress [88]. Furthermore, the development of models capable of distinguishing compounds capable of activating T-cells need to be developed.

### Molecular dynamic simulations of abacavir and acyclovir with co-binding peptide P3

After our initial docking comparison with the proposed HLA-B*57:01 liable compounds from the model employed by Metushi et al. [42], we decided to conduct molecular dynamic simulations to examine why our model did not identify acyclovir as an active drug for HLA-B*57:01 in complex with peptide P3. Using the crystal structure 3UPR, we conducted 20 ns simulations of HLA-B*57:01 with either abacavir or acyclovir and co-binding peptide P3 in a TIP3P water environment (see “Methods”). We selected the peptide P3 for two reasons: (1) the binding mode of abacavir with P3 is explicitly known in a crystal structure (PDB: 3UPR) and (2) Metushi et al.’s [42] finding demonstrated that the binding affinity of P3 for HLA-B*57:01 was significantly enhanced in the presence of acyclovir.

**Fig. 9:**
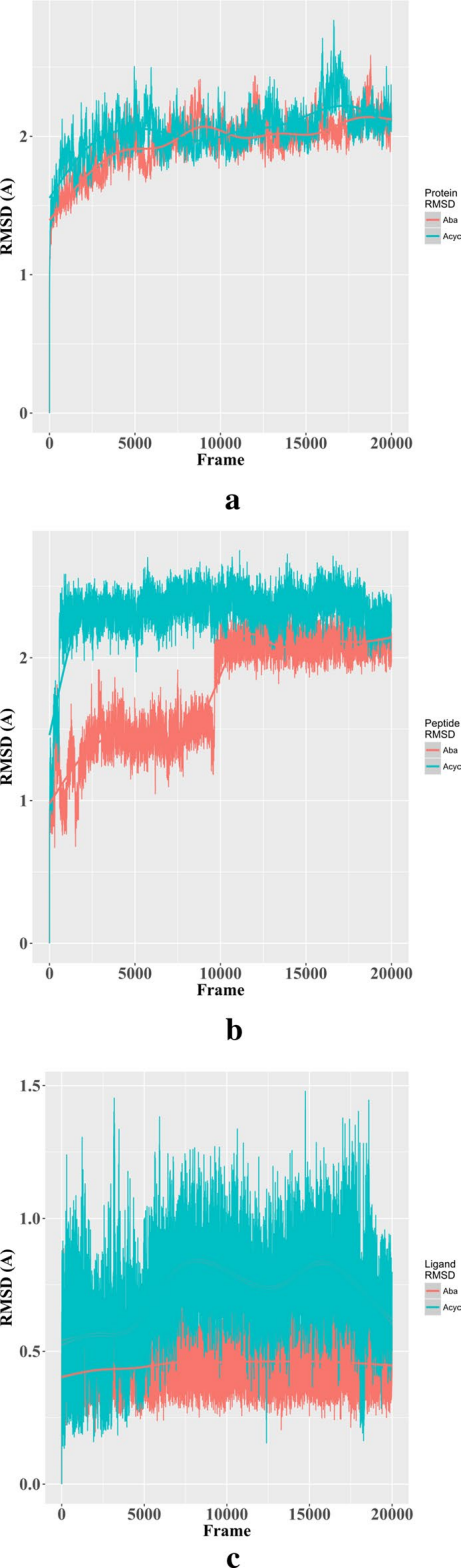
Measured RMSD for 20 ns molecular dynamic simulations of abacavir (red) and acyclovir (blue) when complexed with HLA-B*57:01 protein, ligand, and peptide P3 (PDB: 3UPR). **a** RMSD fluctuation of HLA-B*57:01 protein with respect to ligand, **b** RMSD fluctuation of peptide P3 with respect to ligand, **c** ligand fluctuation inside the pocket

It is important to reiterate that these tripartite systems of HLA-drug-peptide are extremely complex to model and the relationships between the individual components is not well understood. As such, we decided to begin investigating the stability of protein, ligand, and peptide by measuring their respective RMSDs along the MD simulations as shown in Fig. 9. Notably, the HLA-B*57:01 protein was not significantly impacted by either abacavir or acyclovir as the overall RMSD for both models was less than 2 Å (Fig. 9a). However, when the fluctuation of peptide P3 was considered, we observed that, when binding with abacavir, the overall flexibility of P3 in the first 10 ns was rather low (RMSD ≤ 1.5 Å); however, the RMSD of P3 increased to 2 Å in the second part of the simulation (Fig. 9b). Meanwhile, peptide P3 was observed to have an almost constant RMSD of 2 Å when acyclovir was present. Finally, we computed the RMSD fluctuations of abacavir and acyclovir in the binding pocket (Fig. 9c). Abacavir was found to be extremely stable in the binding pocket with minimal conformational changes (RMSD ≤ 0.5 Å); however, the observed RMSD of acyclovir ranged from 0.5 to 1.5 Å. This larger fluctuation in measured RMSD for acyclovir is caused by the increased rotation of the diethyl-ether functional group, which contains several rotatable bonds. Though there are some discrepancies between the measured RMSDs between abacavir and acyclovir, the overall systems are stable with RMSDs less than 2 Å.

**Fig. 10:**
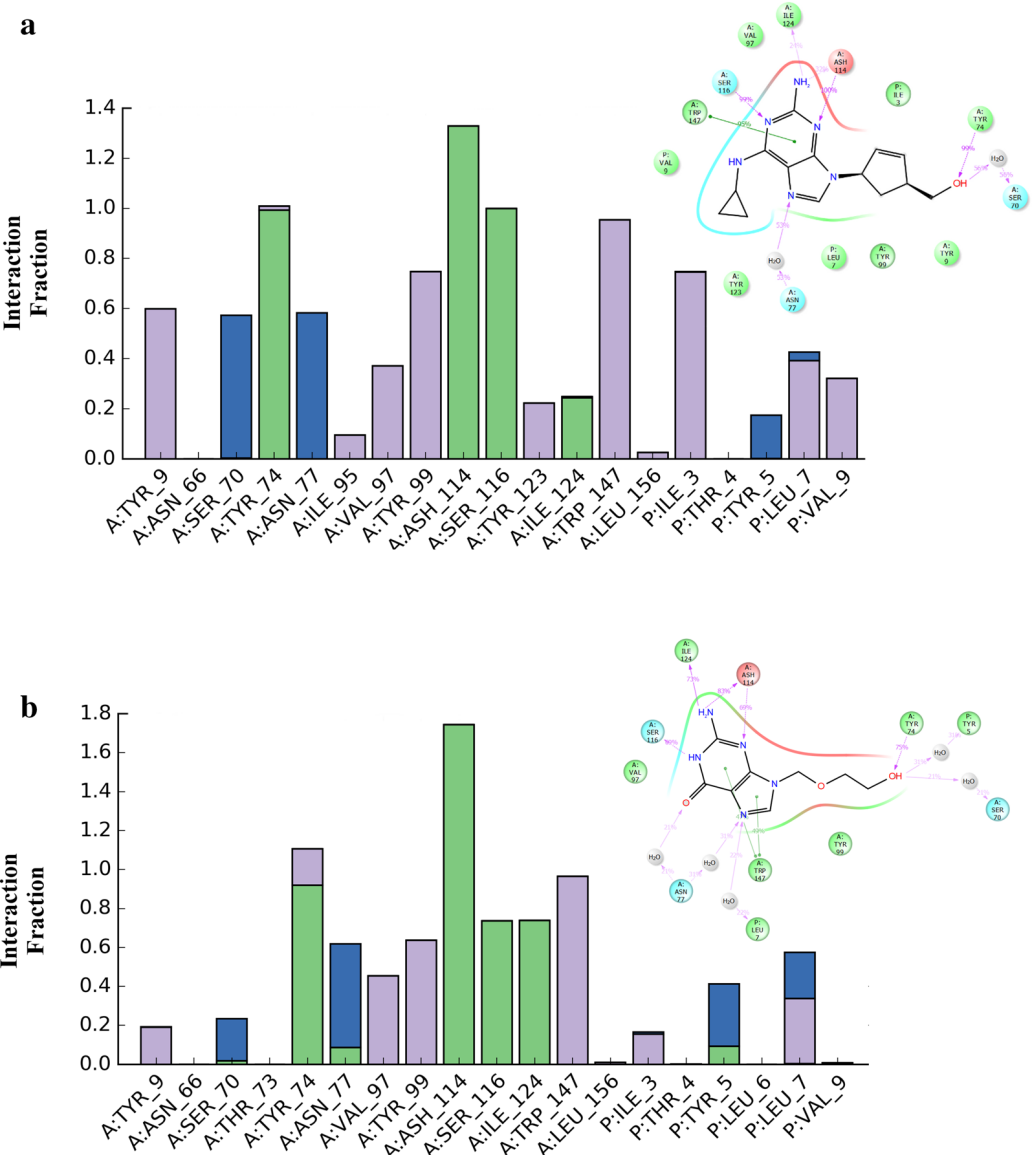
Protein–ligand interaction fragment histograms and 2D-plots for 20 ns molecular dynamic simulation of HLA-B*57:01 with ligand and co-binding peptide P3. **a** Abacavir as ligand, **b** acyclovir as ligand. Hydrogen bond interactions are represented as green bars, water-bridges are blue bars, and hydrophobic interactions (including π–π stacking) are purple bars

Next, we analyzed the time dependencies of drug-protein interactions by comparing binding modes of abacavir and acyclovir with P3 across the entire simulation. Unlike the top-scored binding modes obtained from molecular docking, MD simulations enabled us to (1) analyze all the binding modes by averaging all ligand–protein interactions identified in each frame of the simulation, and (2) determine the most favorable interactions. Figure 10 displays these time-averaged interactions between the binding pocket of 3UPR (chain A) and peptide P3 (labelled chain P) with either abacavir (Fig. 10a) or acyclovir (Fig. 10b) as histogram plots where the x-axis represent the amino acid and the y-axis represents the Interaction Fraction (IF). Additionally, Fig. 10 provides insights into H-bonding (green bars), H-bonding through water-bridges (blue bars), and hydrophobic interactions (purple bars).

Interestingly, abacavir and acyclovir share several key interactions that are conserved throughout the simulation (IF ≥ 0.8). These conserved interactions are H-bonding with residues TYR74, ASH114, SER116 from chain A (binding pocket) and hydrophobic interactions (π–π stacking) with TRP147 also from chain A (Fig. 10a, b). There are some moderately conserved interactions (IF = 0.4–0.6) shared between both simulations with a water bridge formation between ligand and ASN77 and hydrophobic interactions with VAL 97 (both with chain A). Intriguingly, the biggest difference between simulations of abacavir and acyclovir occurred with the ligand-peptide interactions. Abacavir showed very strong hydrophobic interactions with ILE3 of P3 and moderate interactions with LEU7 and VAL9 as shown in Fig. 10a. A weak interaction (IF ≤ 0.3) was observed between TYR5 of P3 and abacavir as well. Intriguingly, no strong interactions were observed between acyclovir and peptide P3, but there were moderate hydrophobic interactions with LEU7 and water-bridge formation with TYR5 of P3 (Fig. 10b). Several weak interactions were observed between acyclovir and P3 including: a weak water bridge with LEU7, weak direct H-bond formation with TYR5, and weak hydrophobic interactions with ILE3.

MD simulations can provide valuable insights into the binding mode stability and favored dynamic ligand–protein interactions. Clearly, the simulations conducted in this study demonstrate that abacavir affords an increased stabilization from peptide P3 resulting in a more stable conformation and lower DS (and eM) than acyclovir. While these insights explain why our docking model identified acyclovir as HLA-B*57:01 inactive, it does not explain why the experimental findings by Metushi et al. [42] indicate acyclovir is HLA-B*57:01 liable with peptide P3. However, this disagreement could occur due to several different factors. First, our molecular docking platform uses two empirical thresholds for DS and eM that have previously been determined to accurately predict ligand binding [69, 70], that we validated using a limited number of test molecules [44]. From this test, there was only one fully solved binding mode of an HLA-drug complex available (abacavir) and two other proposed HLA-B*57:01 active compounds (flucloxacillin and pazopanib) from the use of odds ratios. Building any predictive model with limited experimental evidence, such as HLA-induced ADR models, severely limits the model’s reliability and applicability domain. Therefore, the use of any empirical scoring thresholds needs to be constantly reevaluated as new experimental data emerges. Indeed, virtual screening of large chemical database can provide valuable guidance to experimentalists for the prioritization of drugs to test for HLA-B*57:01 binding and T-cell activation. Such experimental studies could assist in confirming, lowering, or increasing our model’s threshold for selecting the *predicted*-*to*-*be*-*active* molecules. Second, our MD simulation of acyclovir with peptide P3 demonstrated that the formation between HLA-B*57:01, acyclovir, and peptide P3 was stable; however, our docking procedure was based on a rigid (SP) or semi-flexible (XP) protein and peptide. Therefore, it is likely that allowing peptide’s full flexibility and/or employing an ensemble docking technique (using multiple protein conformations) may be necessary to reevaluate fringe compounds (compounds within 1 kcal/mol of our DS threshold). Third, the assay employed by Metushi et al. [42] monitored the binding affinity of the peptide towards the HLA, not the actual binding affinity of the drug. Our molecular docking platform explored the binding association of various drugs inside the binding pocket, but did not analyze the peptide’s binding affinity for these drugs. The development of a peptide-specific molecular docking platform could provide complementary insights into the complex binding relationship between HLA-protein, drug, and co-binding peptides.

## Conclusions and future work

**Fig. 11:**
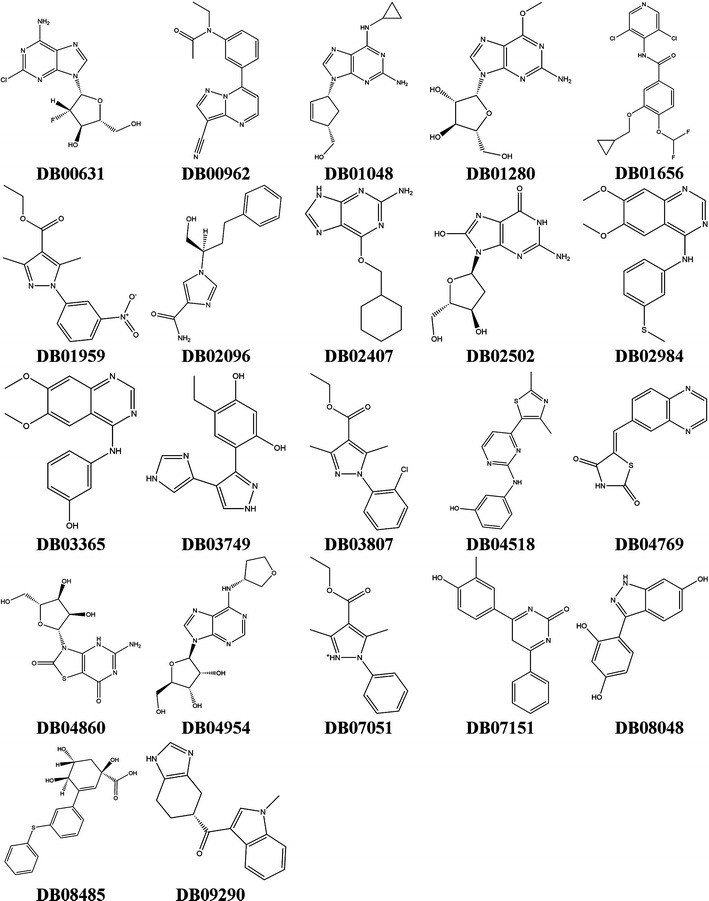
Structures of the 22 active drugs identified from DrugBank screen

Using our multi-peptide, abacavir-specific, consensus docking protocol for the HLA-B*57:01 variant [44], we have screened the whole DrugBank database [47] containing over 7000 drugs and drug candidates. After docking based on two scoring functions, three X-ray crystals 3VRI, 3VRJ, and 3UPR with and without their associated co-binding peptides P1, P2, and P3, respectively, we identified 22 potentially HLA-B*57:01 liable compounds. The chemical scaffolds of these 22 compounds are provided in Fig. 11, while DS are available in Table 2 (eM scores are available in Additional file 1: Table 2). Additionally, our platform could be extended to a 4-tiered approach using the recently solved X-ray crystal structure of HLA-B*57:01 with bound abacavir in the presence of a new co-binding peptide, P4 [19].

After identifying those 22 potential actives, hierarchical clustering was performed using 3D interaction fingerprints from the binding modes of abacavir with peptides P1, P2, or P3. These clustering results revealed three top drug candidates: DB01280 (*nelarabine*), DB02407, and DB04860 (*isatoribin*e). However, clustering revealed that these drugs were not necessarily the top drug candidate for every peptide. Indeed, clustering with P2 revealed no other drugs clustered with abacavir, while clustering with P3 indicated that the drugs DB00962 and DB04954 were the top candidates. Furthermore, it was determined that each screening with peptide P1, P2, or P3 resulted in a different drug being most dissimilar from abacavir. Clearly, the role of co-binding peptide will need to be investigated further to elucidate its role in signaling ADRs.

Using these 22 predicted HLA-B*57:01 liable compounds, we plan to collaborate with experimentalists for the development of an efficient and accurate screening assay for T-cell activation to confirm our model’s predictive capabilities. One possible assay for consideration is the radio-labelled competitive peptide binding assay used by Metushi et al. [42] and the T-cell activation assay developed by Lucas et al. [43]. Notably, as discussed in “Model comparisons to Metushi et al.”, our docking protocol identified 22 new potentially HLA-B*57:01 compounds with only the drug nelarabine (DB01280) overlapping with the Metushi et al. study [42]. Once experimental binding data has been collected, we will continue to refine our ensemble docking protocol for improved prediction accuracy, while simultaneously developing a quantitative structure activity relationship (QSAR) model for the prediction of ADR events that are mediated by a drug’s ability to bind the HLA-B*57:01 variant. Additionally, we performed some preliminary MD simulations to investigate the differences between abacavir and acyclovir when complexed with peptide P3. These initial findings revealed that both abacavir and acyclovir were stable in the HLA-B*57:01 binding pocket, but had significantly different ligand–protein interactions with peptide P3. Future MD simulations will be conducted to elucidate the dynamic intermolecular interactions between the HLA-B*57:01 binding pocket, the different co-binding peptides (P1, P2, P3, and P4), and abacavir, all forming challenging tripartite complexes. There is also a need to explore molecular docking’s capability to accurately score and rank peptide binding modes with HLA-drug complexes to address the diverse number of possible co-binding peptides. Lastly, this study underlines the need of developing a pan-HLA virtual screening workflow incorporating at least 50 variants being the most relevant and frequent in the global populations. This panel of virtual HLA pockets will serve a dual purpose by further exploring drug and HLA binding promiscuity, as observed with the drug carbamazepine and the HLA-A*31:01 and -B*15:02 variants, and developing a co-binding peptide in silico library that determines the most likely HLA-peptide pairings. Conducting such virtual screening studies will provide new insight and guidance for experimentalists attempting to test a drug’s likelihood of inducing ADR events. In return, new experimental data will provide new information for the creation of more sophisticated in silico models and the advancement of Precision Medicine.

## Additional file

**Additional file 1.** Contains tables showing eM scores **(ST1)** and measured Tanimoto similarities scores for 22 predicted DrugBank HLA-B*57:01 liable compounds using interaction fingerprint descriptors (**ST2**). Figures showing Pearson correlations for eM docking conditions, hierarchical clustering in presence of peptide P2 and P3 using interaction fingerprint descriptors, superimpositions and binding modes of select drugs, and measured eM box plot distribution of Metushi et al. compound **(SF1–SF6)**.

## Abbreviations

ADR: adverse drug reaction
WHO: World Health Organization
CYP: cytochrome P450
G6PD: glucose-6-phosphate dehydrogenase
NUDT15: nucleoside diphosphate linked moiety X-type motif 15
ABC: ATP-binding cassette
SLCO1B1: solute carrier organic anion transporter family
APC: antigen presenting cells
HLA: human leukocyte antigen
RVAQLEQVYI: peptide P1
LTTKLTNTNI: peptide P2
HSITYLLPV: peptide P3
DS: Docking Score
eM: eModel Score
MD: molecular dynamics
T_2D_: 2D-Tanimoto similarity
T_IF_: 3D protein–ligand interaction fingerprint Tanimoto similarity
M1: KVAKVEPAV, M2: RVAGIHKKV, M3: HSITYYLPV: peptides from Metushi et al. study
QSAR: quantitative structure activity relationship
