# Supplementary material for: Adverse drug reactions triggered by the common HLA-B*57:01 variant: virtual screening of DrugBank using 3D molecular docking
Source: J Cheminform. 2018 Jan 30;10:3. doi: 10.1186/s13321-018-0257-z (PMC5790764; doi:10.1186/s13321-018-0257-z)
Supplement: Supplementary file 1 — Additional file 1. Contains tables showing eM scores (ST1) and measured Tanimoto similarities scores for 22 predicted DrugBank HLA-B*57:01 liable compounds using interaction fingerprint descriptors (ST2). Figures showing Pearson correlations for eM docking conditions, hierarchical clustering in presence of peptide P2 and P3 using interaction fingerprint descriptors, superimpositions and binding modes of select drugs, and measured eM box plot distribution of Metushi et al. compound (SF1–SF6). [file 13321_2018_257_MOESM1_ESM.docx]

**
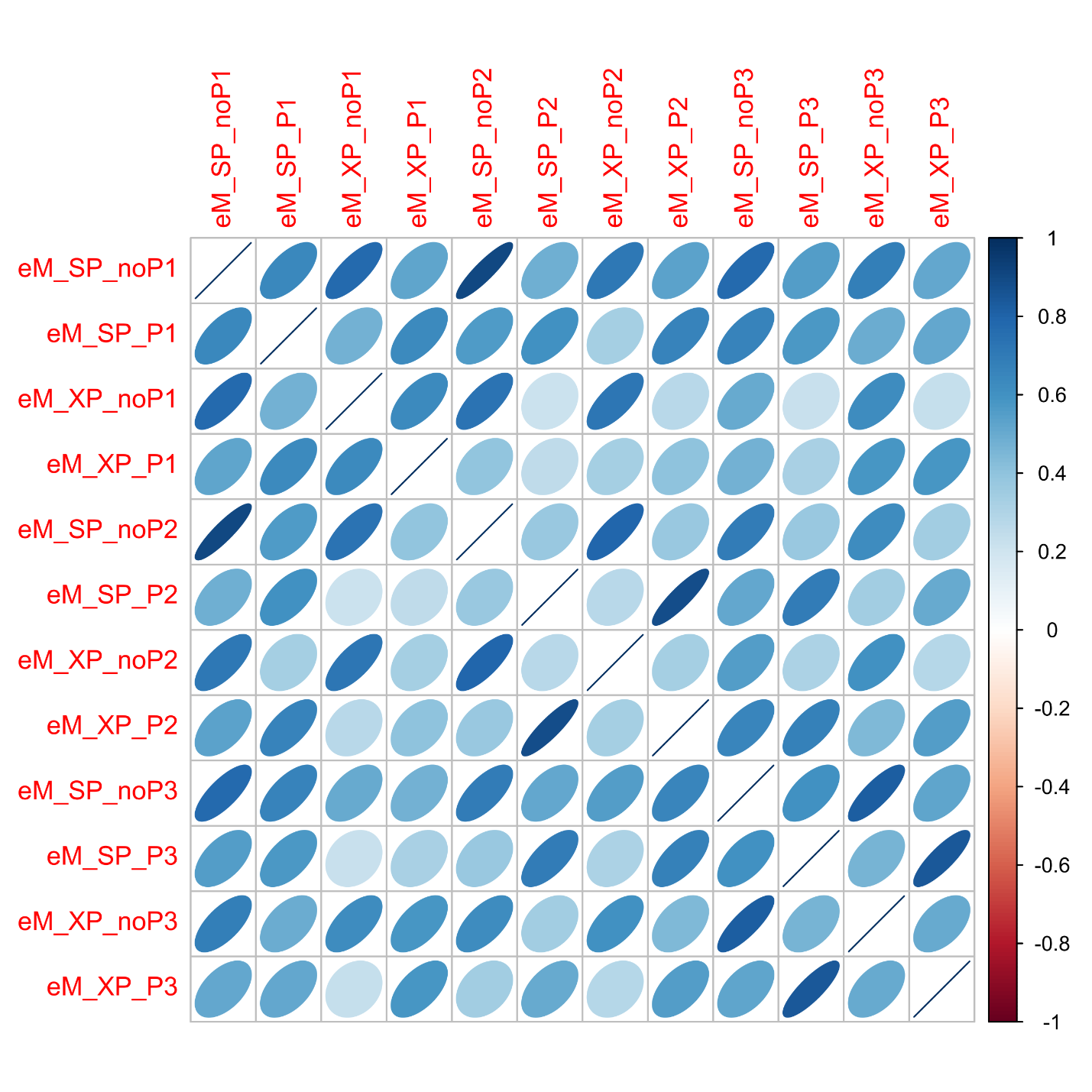
**

**Supplementary Figure 1**. Heat map representation of all pairwise Pearson correlation coefficients between all eModel (eM) scores.


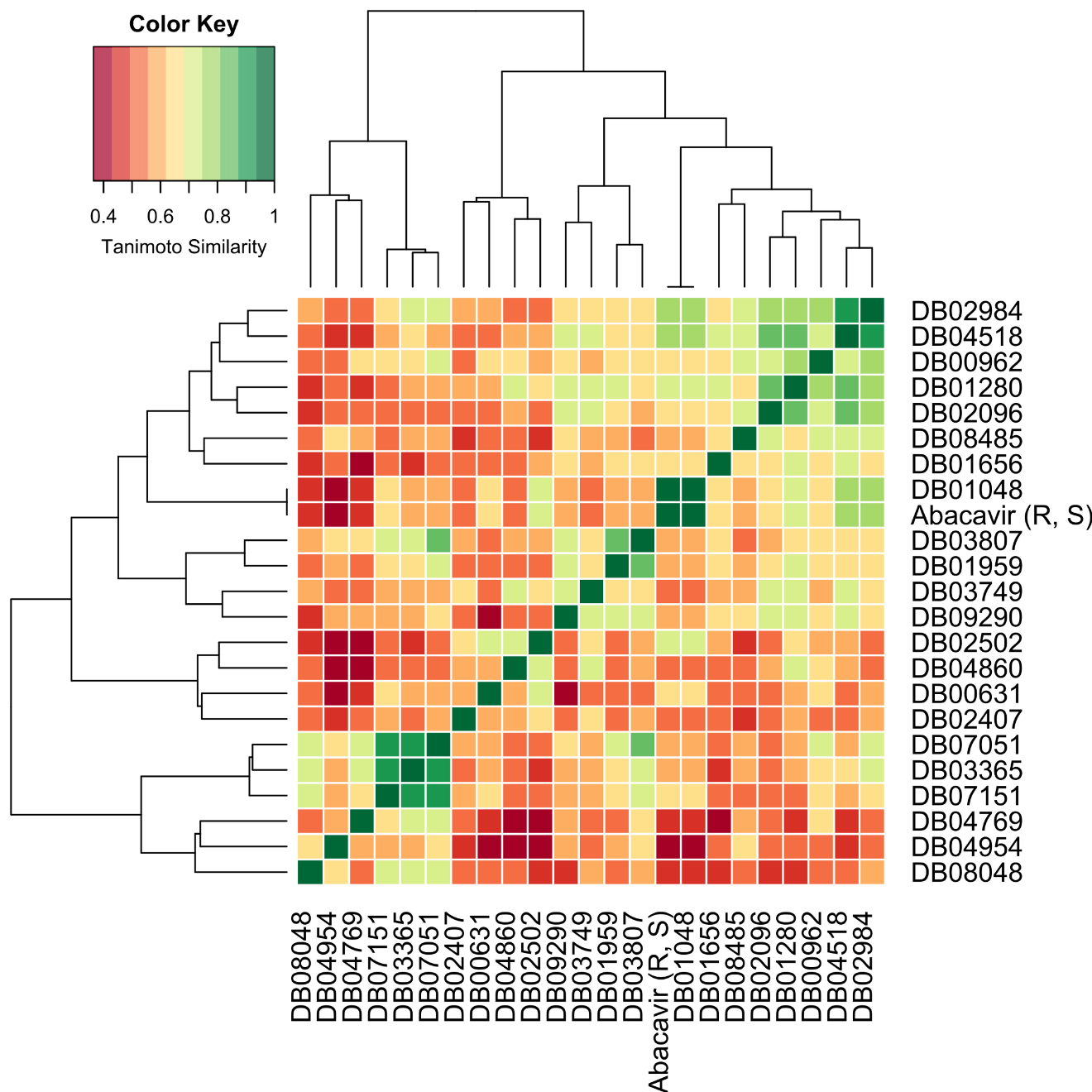


**Supplementary Figure 2**. Heat map representation of the interaction fingerprint similarity matrix clustered using the Ward algorithm. Docking was conducted in presence of peptide P2. Red cells indicate a low Tanimoto similarity (<0.3), yellow cells represent moderate Tanimoto similarity (0.3-0.7), and green cells indicate high Tanimoto similarity (>0.7).

**
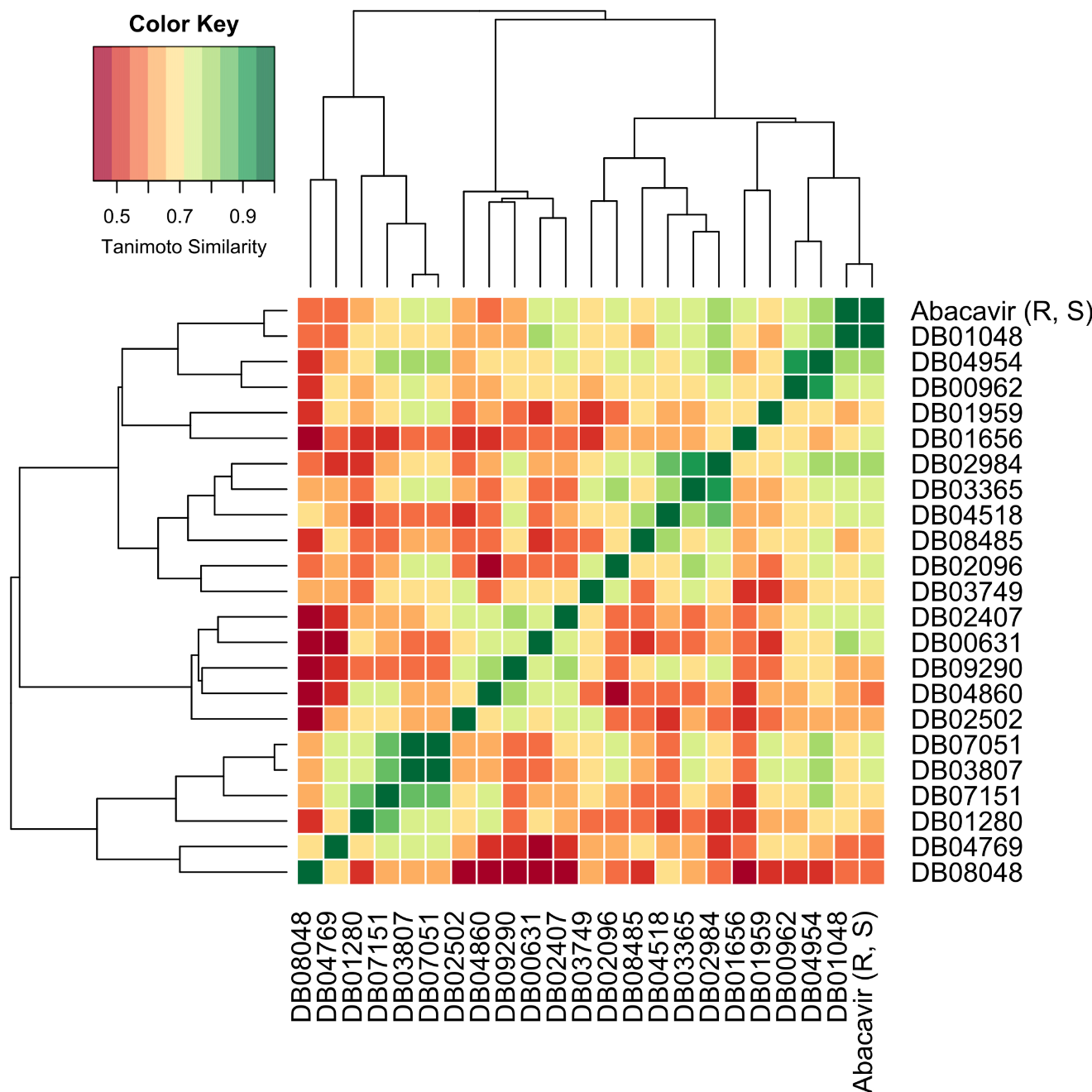
**

**Supplementary Figure 3**. Heat map representation of the interaction fingerprint similarity matrix clustered using the Ward algorithm. Docking was conducted in presence of peptide P3. Red cells indicate a low Tanimoto similarity (<0.3), yellow cells represent moderate Tanimoto similarity (0.3-0.7), and green cells indicate high Tanimoto similarity (>0.7).

| 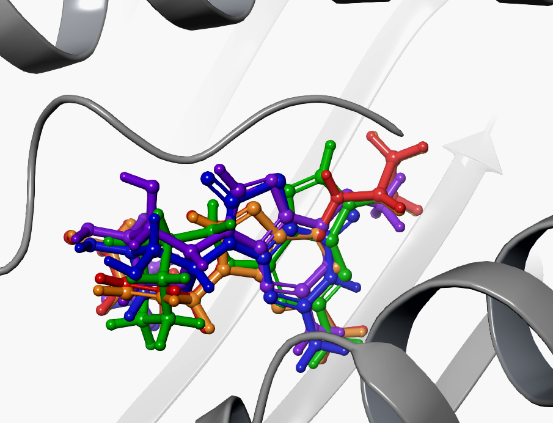 | 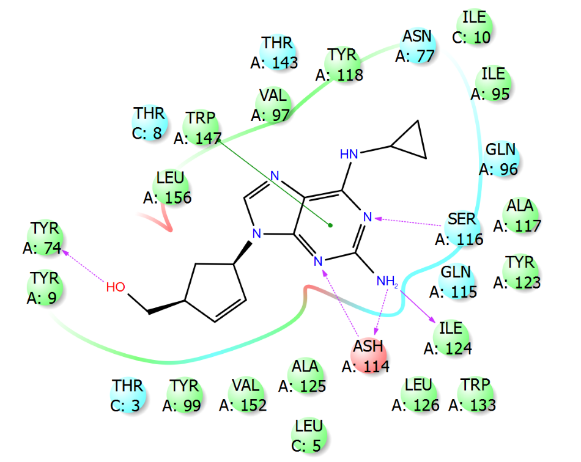  **Native Abacavir (3VRJ)** | |
| --- | --- | --- |
|  |  |  |
| **A** | **B** | |
| 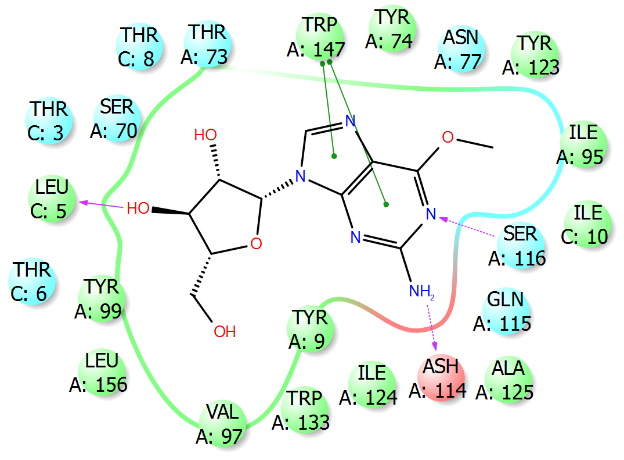  **DB001280 (**DS: -8.9; eM: -75.0) | 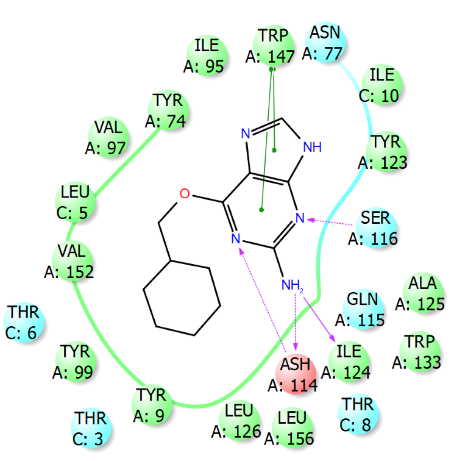  **DB02407 (**DS: -7.4; eM: -67.4) | |
| **C** | **D** | |
| 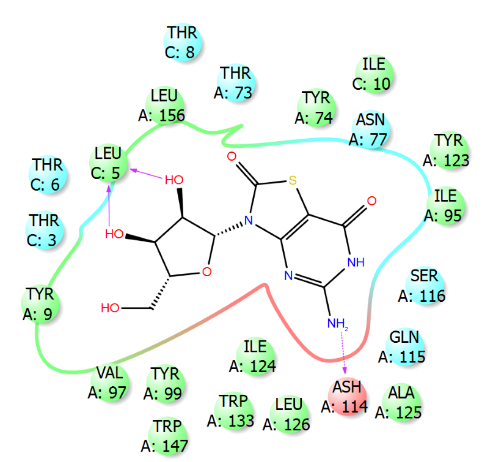  **DB04860 (**DS: -10.5; eM: -64.6) | | |
| **E** | | |

**Supplementary Figure 4. (A)** Superimposition of a cluster of five drugs: Abacavir (*red*), DB01048 (abacavir from DrugBank, *orange*), DB01280 (*purple*), DB02407 (*green*), and DB04860 (*blue*) using their poses from the (XP + P2) docking results. Binding modes of **(B)** native abacavir (PDB: 3VRJ), **(C)** DB01280, **(D)** DB02407, and **(E)** DB04860 with their associated DS and eM scores.

| 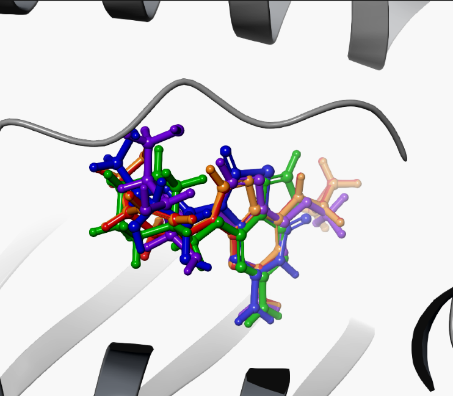 | 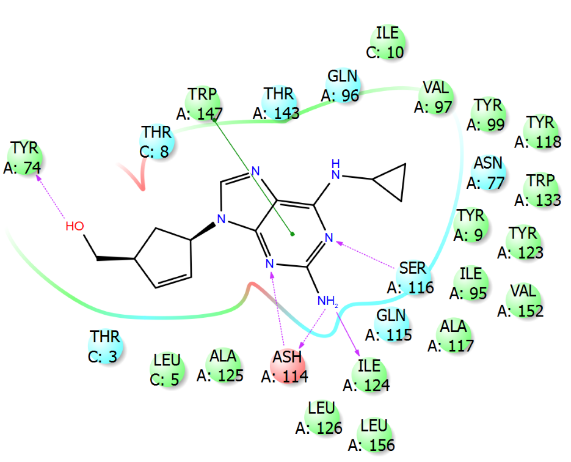  **Native Abacavir (3UPR)** | |
| --- | --- | --- |
|  |  |  |
| **A** | **B** | |
| 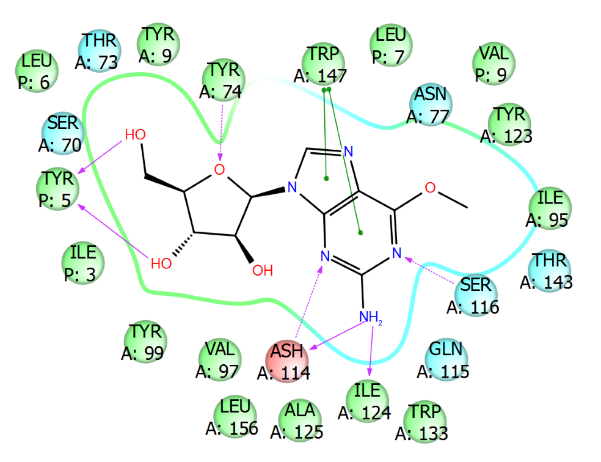  **DB001280** (DS: -10.2; eM: -62.0) | 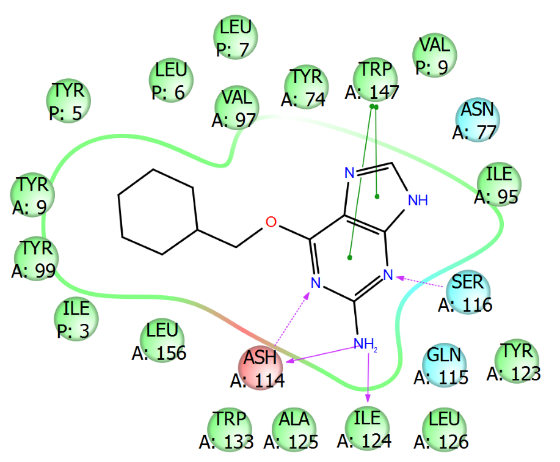  **DB02407** (DS: -7.8; eM: -63.7) | |
| **C** | **D** | |
| 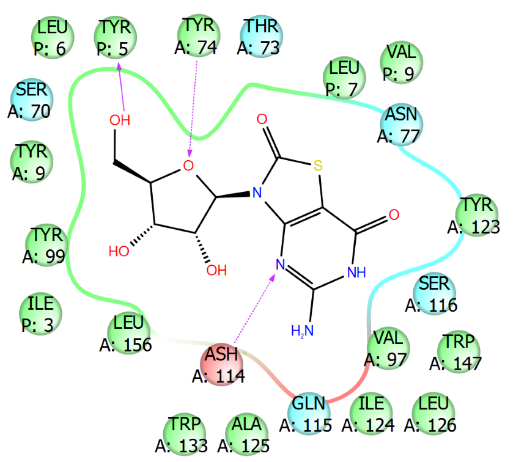  **DB04860** (DS: -11.2; eM: -53.2) | | |
| **E** | | |

**Supplementary Figure 5. (A)** Superimposition of a cluster of five drugs: Abacavir (*red*), DB01048 (abacavir from DrugBank, *orange*), DB01280 (*purple*), DB02407 (*green*), and DB04860 (*blue*) using the poses from (XP + P3) docking results. Binding modes of **(B)** native abacavir (PDB: 3UPR), **(C)** DB01280, **(D)** DB02407, and **(D)** DB04860 with their associated DS and eM scores from the (XP + P3) docking.


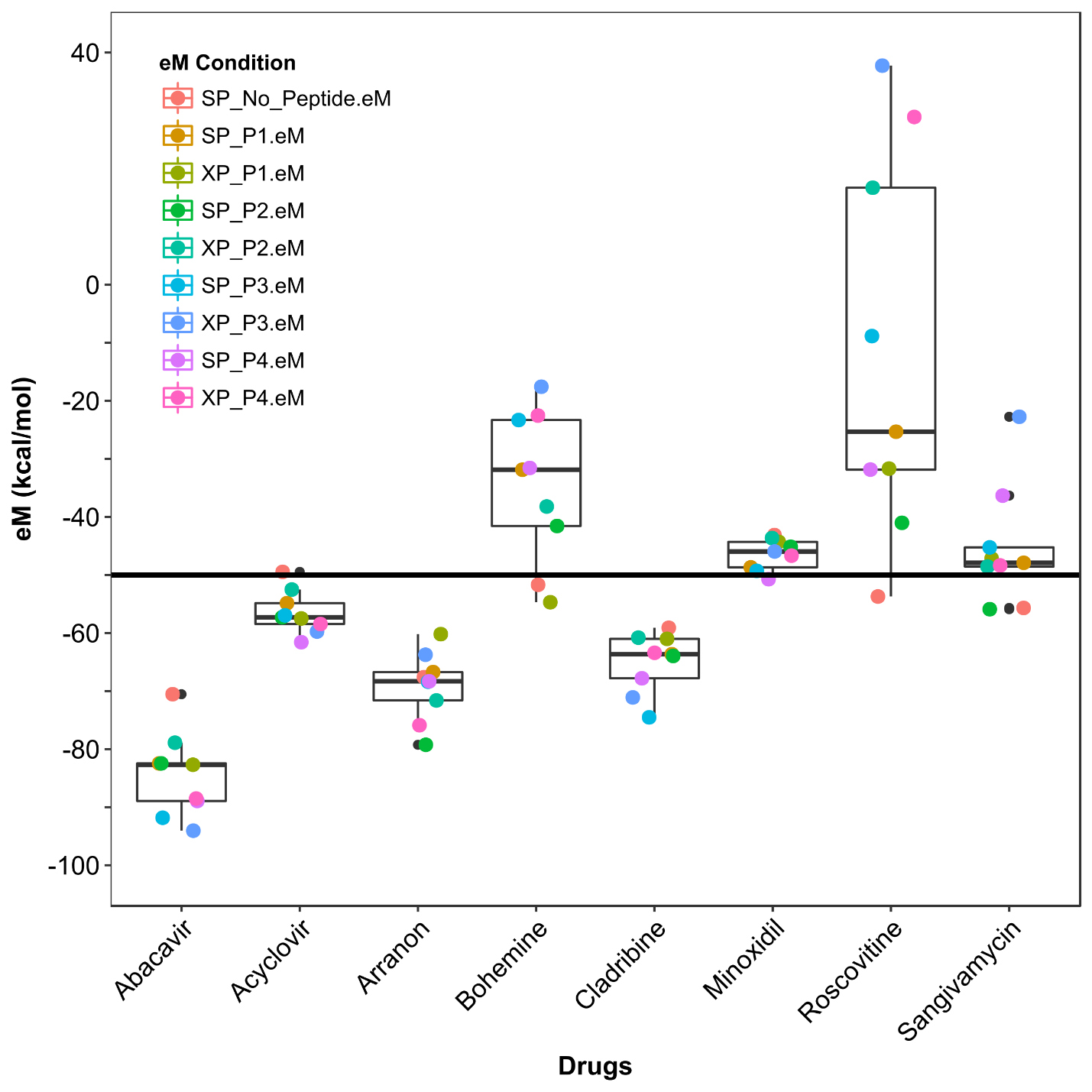


**Supplementary Figure 6.** Glide eM scores for abacavir (DB01048) and seven proposed HLA-B*57:01 active compounds identified by Metushi et al. from the ZINC database. Those seven compounds are: Acyclovir (DB00787), arranon (DB01280 or nelarabine), bohemine, cladribine (DB00242), minoxidil (DB00350), roscovitine, and sangivamycin. All eM scores are visualized as boxplots with superimposed, 1D-vertical scatter plots with applied horizontal jitter to prevent data point overlap. Each data point is color coded per the condition of docking: SP without peptide (*salmon*), PDB: 3VRI), SP with P1 (*gold*), XP with P1 (olive *green*), SP with P2 (*green*), XP with P2 (*turquoise*), SP with P3 (*light blue*), XP with P3 (*blue*), SP with P4 (*purple*), and XP with P4 (*pink*). The eM threshold score (eM < -50 kcal/mol) is marked as a black line on the plot.

**Supplementary Table 1.** Recapitulative DS scores obtained for the 22 hit compounds identified from the screening of DrugBank.

|  | P1 | | | | P2 | | | | P3 | | | |
| --- | --- | --- | --- | --- | --- | --- | --- | --- | --- | --- | --- | --- |
|  | SP | | XP | | SP | | XP | | SP | | XP | |
| DrugBank ID | (−) | (+) | (−) | (+) | (−) | (+) | (−) | (+) | (−) | (+) | (−) | (+) |
| DB00631 | -7.4 | -8.3 | -7.2 | -8.1 | -7.4 | -9.3 | -7.2 | -8.6 | -7.9 | -9.5 | -8.0 | -8.0 |
| DB00962 | -7.3 | -9.8 | -7.2 | -9.5 | -7.9 | -9.6 | -7.8 | -8.0 | -8.9 | -8.8 | -8.5 | -9.1 |
| DB01048 | -8.6 | -10.4 | -8.4 | -9.6 | -8.0 | -9.8 | -7.3 | -9.2 | -8.3 | -10.6 | -7.9 | -10.1 |
| DB01280 | -8.0 | -8.5 | -8.6 | -9.3 | -8.1 | -9.9 | -8.1 | -8.9 | -7.3 | -9.6 | -8.6 | -10.2 |
| DB01656 | -7.6 | -8.4 | -8.8 | -9.8 | -7.1 | -8.5 | -8.4 | -9.7 | -7.9 | -8.1 | -7.4 | -9.4 |
| DB09290 | -7.5 | -7.4 | -8.0 | -10.2 | -7.3 | -8.8 | -8.0 | -8.4 | -8.6 | -8.3 | -8.6 | -8.8 |
| DB04860 | -7.1 | -9.8 | -7.5 | -9.4 | -7.2 | -8.6 | -7.4 | -10.5 | -8.3 | -9.5 | -8.9 | -11.2 |
| DB04954 | -7.2 | -9.0 | -8.7 | -9.6 | -7.7 | -9.5 | -8.2 | -11.0 | -8.0 | -9.4 | -8.7 | -10.5 |
| DB01959 | -8.5 | -9.9 | -8.2 | -9.0 | -8.7 | -8.8 | -8.1 | -8.6 | -9.0 | -9.1 | -8.4 | -9.1 |
| DB02096 | -7.1 | -8.5 | -8.1 | -9.9 | -7.6 | -8.3 | -8.3 | -9.2 | -7.2 | -7.9 | -8.1 | -9.1 |
| DB02407 | -8.3 | -9.2 | -8.4 | -9.2 | -8.4 | -7.8 | -8.1 | -7.4 | -8.5 | -7.0 | -8.1 | -7.8 |
| DB02502 | -7.3 | -8.7 | -7.7 | -9.8 | -7.6 | -8.2 | -7.8 | -9.0 | -8.4 | -8.1 | -8.1 | -10.3 |
| DB02984 | -8.5 | -10.0 | -8.8 | -10.6 | -8.7 | -8.0 | -8.9 | -8.3 | -8.5 | -8.1 | -8.7 | -8.2 |
| DB03365 | -7.7 | -9.2 | -8.8 | -9.8 | -7.8 | -9.6 | -8.6 | -10.0 | -7.6 | -9.2 | -7.8 | -10.2 |
| DB03749 | -7.5 | -8.3 | -7.6 | -8.6 | -7.1 | -8.4 | -7.5 | -9.0 | -7.4 | -8.1 | -7.7 | -8.9 |
| DB03807 | -8.0 | -9.9 | -7.8 | -10.5 | -8.4 | -9.4 | -8.2 | -8.5 | -8.7 | -8.5 | -8.8 | -9.7 |
| DB04518 | -8.0 | -8.8 | -8.8 | -10.4 | -7.9 | -9.3 | -8.2 | -9.3 | -7.2 | -9.4 | -8.1 | -9.6 |
| DB04769 | -7.5 | -9.3 | -7.1 | -8.9 | -7.1 | -9.8 | -7.3 | -10.1 | -7.9 | -9.7 | -7.3 | -10.2 |
| DB07051 | -8.2 | -9.5 | -7.9 | -9.7 | -8.1 | -8.9 | -8.3 | -9.2 | -8.4 | -8.9 | -8.6 | -9.3 |
| DB07151 | -8.2 | -9.9 | -9.0 | -10.6 | -8.2 | -10.5 | -9.0 | -11.0 | -8.8 | -10.6 | -9.8 | -11.3 |
| DB08048 | -7.1 | -8.1 | -7.6 | -7.8 | -7.4 | -8.8 | -8.0 | -8.3 | -7.6 | -8.2 | -7.9 | -8.8 |
| DB08485 | -7.2 | -8.5 | -10.0 | -11.2 | -7.1 | -8.6 | -9.1 | -7.6 | -8.0 | -9.6 | -10.9 | -11.5 |

**Supplementary Table 2.** Recapitulative eM scores obtained for the 22 hit compounds identified from the screening of DrugBank.

|  | P1 | | | | P2 | | | | P3 | | | |
| --- | --- | --- | --- | --- | --- | --- | --- | --- | --- | --- | --- | --- |
|  | SP | | XP | | SP | | XP | | SP | | XP | |
| DrugBank ID | (−) | (+) | (−) | (+) | (−) | (+) | (−) | (+) | (−) | (+) | (−) | (+) |
| DB00631 | -61.9 | -61.3 | -61.1 | -55.2 | -59.8 | -69.7 | -59.1 | -59.2 | -56.1 | -72.0 | -54.3 | -61.6 |
| DB00962 | -58.7 | -72.8 | -62.1 | -71.0 | -57.2 | -72.6 | -51.4 | -69.4 | -60.3 | -56.7 | -60.2 | -52.9 |
| DB01048 | -69.9 | -81.7 | -66.3 | -77.7 | -64.8 | -82.3 | -62.9 | -82.2 | -70.1 | -91.4 | -68.0 | -95.0 |
| DB01280 | -66.5 | -67.3 | -60.3 | -59.2 | -64.8 | -79.4 | -63.3 | -75.1 | -60.5 | -67.8 | -57.7 | -62.0 |
| DB01656 | -63.2 | -64.0 | -70.0 | -71.2 | -61.8 | -52.9 | -64.1 | -52.8 | -62.1 | -62.5 | -67.4 | -64.9 |
| DB09290 | -59.9 | -61.6 | -54.3 | -56.0 | -59.8 | -60.3 | -56.5 | -57.5 | -60.9 | -58.8 | -60.5 | -54.9 |
| DB04860 | -58.9 | -69.7 | -54.0 | -55.0 | -58.9 | -64.4 | -58.2 | -64.6 | -62.8 | -68.1 | -59.1 | -53.2 |
| DB04954 | -62.0 | -71.0 | -59.9 | -61.8 | -64.6 | -78.5 | -62.4 | -78.2 | -67.1 | -76.5 | -66.9 | -73.2 |
| DB01959 | -69.0 | -72.6 | -68.9 | -68.0 | -70.9 | -71.7 | -70.1 | -72.6 | -65.8 | -72.8 | -64.0 | -73.0 |
| DB02096 | -52.8 | -62.9 | -54.6 | -61.8 | -53.8 | -58.6 | -56.9 | -58.2 | -51.6 | -53.8 | -52.5 | -52.7 |
| DB02407 | -62.8 | -66.5 | -57.6 | -66.5 | -62.1 | -61.8 | -55.9 | -67.4 | -62.8 | -61.3 | -55.8 | -63.7 |
| DB02502 | -55.1 | -70.0 | -50.7 | -60.4 | -56.0 | -62.3 | -56.0 | -57.8 | -57.3 | -62.1 | -52.0 | -62.8 |
| DB02984 | -67.4 | -71.0 | -68.7 | -61.1 | -68.4 | -58.3 | -62.4 | -54.6 | -63.2 | -57.3 | -59.3 | -50.5 |
| DB03365 | -60.0 | -64.2 | -62.5 | -61.7 | -61.5 | -67.4 | -63.1 | -62.3 | -59.8 | -53.4 | -57.7 | -52.4 |
| DB03749 | -57.7 | -65.6 | -60.6 | -60.9 | -63.8 | -63.6 | -60.4 | -53.7 | -57.4 | -60.0 | -55.4 | -50.1 |
| DB03807 | -59.5 | -67.7 | -54.1 | -57.5 | -60.9 | -66.1 | -56.8 | -58.5 | -59.3 | -64.7 | -57.4 | -62.8 |
| DB04518 | -60.3 | -62.0 | -56.6 | -66.1 | -61.9 | -59.5 | -58.6 | -51.7 | -58.6 | -60.5 | -62.9 | -71.5 |
| DB04769 | -53.7 | -61.7 | -51.8 | -57.3 | -53.9 | -63.1 | -51.7 | -56.3 | -55.8 | -63.0 | -52.4 | -56.3 |
| DB07051 | -58.0 | -65.1 | -54.7 | -57.5 | -57.7 | -63.9 | -51.3 | -58.4 | -55.7 | -68.8 | -53.4 | -69.4 |
| DB07151 | -63.7 | -68.5 | -59.1 | -58.5 | -64.3 | -68.7 | -60.8 | -63.6 | -66.5 | -63.8 | -65.1 | -54.5 |
| DB08048 | -53.0 | -55.7 | -55.0 | -53.5 | -52.9 | -60.2 | -56.9 | -58.8 | -56.1 | -56.8 | -55.5 | -50.7 |
| DB08485 | -57.4 | -60.2 | -52.2 | -58.0 | -56.9 | -65.1 | -57.2 | -62.0 | -63.0 | -67.7 | -57.6 | -65.4 |

**Supplementary Table 3.** Computed Tanimoto similarities for the 22 DrugBank hit compounds compared to abacavir using the 3D interaction fingerprint descriptors.

| **DATABASE**  **ID** | **T_IF_** | | |
| --- | --- | --- | --- |
|  | **XP + P1** | **XP + P2** | **XP + P3** |
| DB00631 | 0.24 | 0.68 | 0.75 |
| DB00962 | 0.63 | 0.67 | 0.76 |
| DB01048 | **0.88** | **1.00** | **0.96** |
| DB01280 | 0.79 | 0.70 | 0.65 |
| DB01656 | 0.64 | 0.68 | 0.73 |
| DB01959 | 0.48 | 0.61 | 0.67 |
| DB02096 | 0.65 | 0.66 | 0.74 |
| DB02407 | 0.68 | 0.55 | 0.73 |
| DB02502 | 0.45 | 0.74 | 0.61 |
| DB02984 | 0.60 | 0.76 | **0.82** |
| DB03365 | 0.45 | 0.59 | 0.75 |
| DB03749 | 0.41 | 0.55 | 0.67 |
| DB03807 | 0.50 | 0.60 | 0.73 |
| DB04518 | 0.56 | 0.80 | 0.73 |
| DB04769 | 0.44 | 0.43 | 0.55 |
| DB04860 | 0.75 | 0.55 | 0.59 |
| DB04954 | 0.56 | 0.42 | **0.81** |
| DB07051 | 0.44 | 0.59 | 0.75 |
| DB07151 | 0.65 | 0.68 | 0.67 |
| DB08048 | 0.30 | 0.48 | 0.55 |
| DB08485 | 0.56 | 0.58 | 0.67 |
| DB09290 | 0.51 | 0.56 | 0.64 |
